# Bacterial Capture‐Killing Capsules with Remodeling Bone Immune Microenvironment for the Effective Treatment of Osteomyelitis

**DOI:** 10.1002/advs.202501505

**Published:** 2025-04-07

**Authors:** Dong Yang, Chang Shu, Chengwei Xu, Xingjie Zan, Shaoyin Wei, Chenglong Wang, Lianxin Li

**Affiliations:** ^1^ School of Ophthalmology and Optometry Eye Hospital School of Biomedical Engineering Wenzhou Medical University Wenzhou Zhejiang 325035 China; ^2^ Wenzhou Medical University Yongkang First People's Hospital Jinhua Zhejiang 321300 China; ^3^ Department of Orthopedics The First Affiliated Hospital of Wenzhou Medical University Wenzhou Zhejiang 325000 China; ^4^ Wenzhou Institute University of Chinese Academy of Sciences Wenzhou Zhejiang 325001 China; ^5^ Key Laboratory of Colloid and Interface Chemistry of the Ministry of Education School of Chemistry and Chemical Engineering Shandong University Jinan Shandong 250100 China; ^6^ Department of Orthopaedics Surgery Shandong Provincial Hospital Affiliated to Shandong First Medical University Jinan Shandong 250021 China

**Keywords:** antibacterial, bone regeneration, microenvironment, osteomyelitis, procyanidins capsule

## Abstract

Osteomyelitis represents a significant health concern, characterized by a bacterial infection that can potentially present a considerable challenge to clinical treatment. Current treatment strategies, including prolonged antibiotic regimens and surgical debridement, often fail to adequately resolve infection or support bone regeneration, largely due to the pathogen‐induced dysregulation of the bone microenvironment. This study reports a multifunctional capsule that achieves coordinated bacterial capture‐killing and immunomodulatory within osseous tissue, effectively resolving the dual pathological challenges of persistent infection microenvironment and dysregulated bone regeneration inherent to osteomyelitis. The capsules, PC‐O@TOB, are based on the Schiff base reaction of procyanidins (PC) with the amino of Lysine6‐osteogenic growth peptide (K6‐OGP) and tobramycin (TOB), which allows for efficient loading and controllable release of K6‐OGP and TOB. In vitro and in vivo studies demonstrated that PC‐O@TOB exhibits dual functionality: potent bactericidal activity through bacterial capture and localized antibiotic delivery, and microenvironmental remodeling via ROS scavenging and M2 macrophage polarization. This immunomodulatory effect synergizes with the capsule's osteogenic and angiogenic properties to accelerate bone repair. The strategy based on antibacterial and later bone microenvironment remodeling has opened up a new way for the treatment of osteomyelitis.

## Graphical abstract

The PC‐O@TOB capsules are designed to exhibit bactericidal effects, regulate the bone immune microenvironment by promoting M2 macrophage polarization, eliminate excessive ROS, and enhance bone regeneration, providing a novel multi‐dimensional approach for the treatment of osteomyelitis.

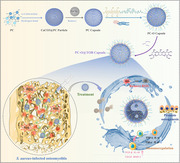

## 1 Introduction

Osteomyelitis, a severe bone tissue disease caused by bacterial infection, is relatively common in patients after surgery or with open fractures.^[^ ^1^, ^2^ ^]^ It is caused by microbial infection of deep bone tissue and is characterized by stubbornness and recurrence.^[^ ^3^, ^4^ ^]^ High recurrence rate and difficulty in repairing bone defects are the main treatment challenges of this disease. Although modern medical technology has reduced the incidence of osteomyelitis to a certain extent, there are still about one million new cases worldwide each year, and the recurrence rate is as high as 20% to 30%, which poses a great challenge to clinical treatment.^[^ ^5^ ^]^ Current standard care protocols combining prolonged high‐dose antibiotics with surgical debridement demonstrate suboptimal efficacy in achieving microbial eradication and functional bone restoration.^[^ ^6^, ^7^ ^]^

Once bacteria invade the bone, the body's defense mechanism will be rapidly activated, thereby triggering a strong inflammatory response.^[^ ^8^, ^9^ ^]^ Among them, macrophages play a key role and polarize into two types, M1‐type macrophages and M2‐type macrophages, according to different stimulus signals.^[^ ^10^, ^11^ ^]^ Under the stimulation of bacterial infection and inflammation, M1‐type macrophages are massively activated and release pro‐inflammatory factors, such as tumor necrosis factor‐α (TNF‐α) and interleukin‐1 (IL‐1). These factors will attract more immune cells to participate in the inflammatory response and further release pro‐inflammatory factors, thus forming a vicious cycle of pro‐inflammatory factor‐M1 activation. M2‐type macrophages, on the other hand, play a role in the later stage of inflammation or under specific regulatory mechanisms and participate in tissue repair and remodeling by secreting growth factors and anti‐inflammatory cytokines. However, during the pathological process of osteomyelitis, the inflammatory response is often difficult to be controlled effectively, making it hard to utilize the repair function of M2‐type macrophages to the fullest extent.^[^ ^12^ ^]^ Concurrent redox dysregulation exacerbates tissue injury, as reactive oxygen species (ROS) surpass bactericidal thresholds to induce lipid peroxidation, proteotoxic stress, and DNA damage ultimately triggering osteonecrosis.

Sterilization of infectious foci remains the therapeutic linchpin.^[^ ^13^ ^]^ Localized delivery of antimicrobial agents achieves high drug concentrations at the infection site, markedly enhancing antibacterial efficacy.^[^ ^14^, ^15^, ^16^ ^]^ Nano‐/micro‐particles further optimize drug loading and controlled release, ensuring sustained therapeutic action,^[^ ^17^ ^]^ while their inherent mobility facilitates bacterial contact and eradication—particularly crucial for deep‐seated infections like tibial osteomyelitis.^[^ ^18^ ^]^ Crucially, post‐sterilization immune microenvironmental regulation is pivotal for bone regeneration.^[^ ^19^, ^20^ ^]^ Persistent inflammation disrupts macrophage polarization balance, impairing tissue repair.^[^ ^21^ ^]^ To counteract these pathological changes, modulating bone immunity (through M2 macrophage activation) and scavenging excessive ROS constitute critical interventions for bone microenvironment regulation. ROS overload alters bone metabolic homeostasis, manifesting as both suppressed osteoblast activity with diminished bone formation and enhanced osteoclast‐mediated resorption.^[^ ^22^, ^23^ ^]^ Thus, effective treatment requires not only pathogen eradication but also rapid establishment of a pro‐regenerative microenvironment.^[^ ^24^ ^]^ Despite its clinical significance, current osteomyelitis research predominantly focuses on antibacterial strategies, with scant attention to post‐sterilization immune microenvironmental regulation.^[^ ^25^ ^]^

**Scheme 1:**
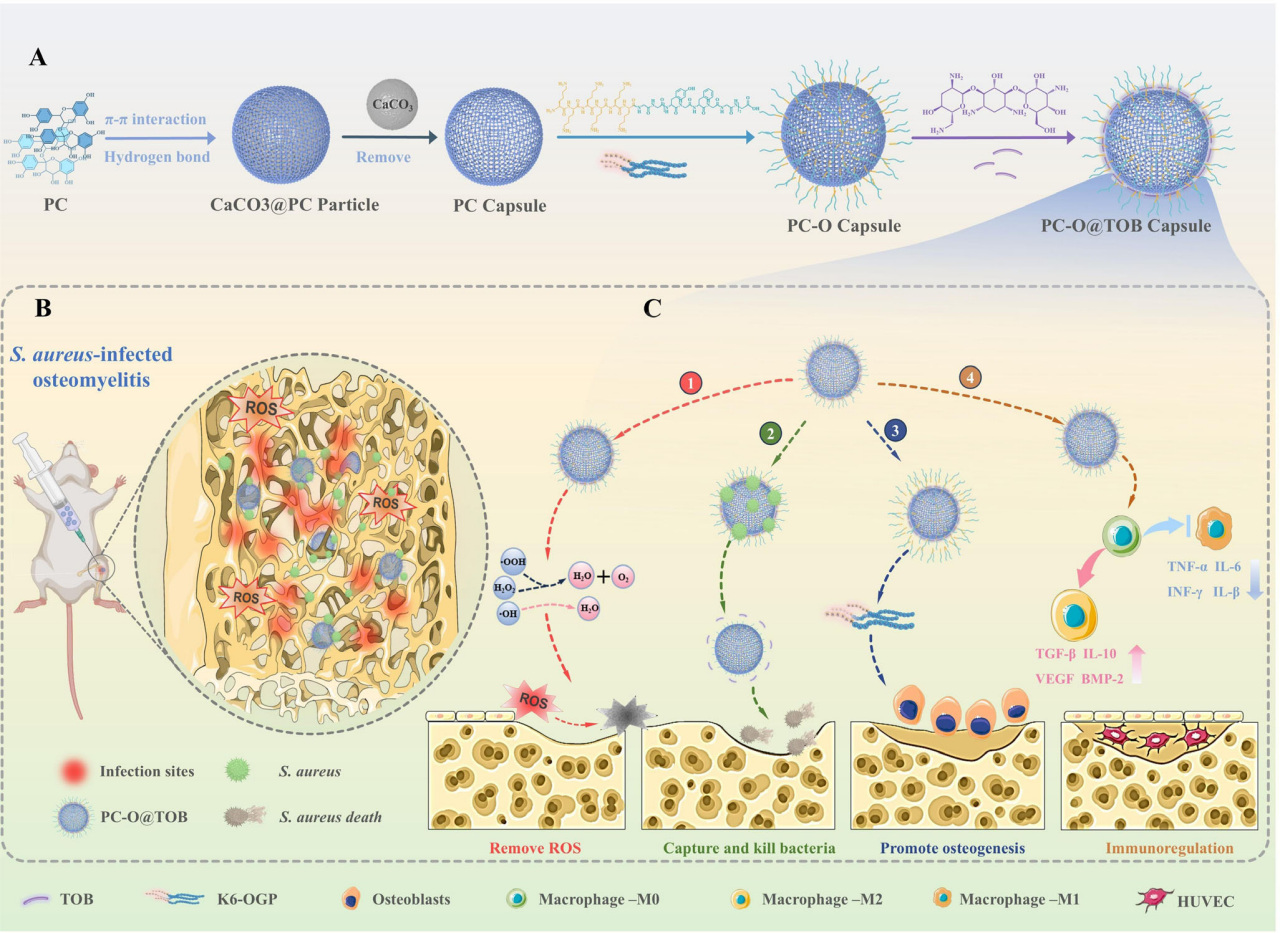
A) Schematic illustration of preparing PC‐O@TOB capsules. PC were self‐assembled onto calcium carbonate (CaCO₃) templates via synergistic π‐π stacking and hydrogen bonding, yielding CaCO₃@PC core‐shell particles. Subsequent removal of CaCO₃ templates afforded hollow PC capsules, which were stepwise functionalized through Schiff base reactions: initial conjugation with K6‐OGP generated PC‐O capsules, followed by TOB loading to produce PC‐O@TOB capsules. B) *S. aureus*‐induced osteomyelitis model. PC‐O@TOB capsules were locally administered to the infected tibial region for targeted anti‐infective therapy. C) Multimodal therapeutic mechanisms of PC‐O@TOB capsules against osteomyelitis: ① ROS to alleviate oxidative stress;② Trapping and eliminating bacteria for effective infection control; ③ Sustained release of K6‐OGP to stimulate osteogenesis and bone regeneration; ④ Immunomodulation via macrophage polarization, reshaping the immune microenvironment to accelerate healing.

In a previous report, we found that plant‐derived PC capsules have remarkable antioxidant and anti‐inflammatory properties.^[^ ^26^ ^]^ PC capsules can significantly improve the stability and bioavailability of PC and achieve the repair of arthritis and diabetic wounds through a single administration.^[^ ^26^, ^27^ ^]^ While demonstrating efficacy in chronic inflammatory contexts, their therapeutic potential for osteomyelitis demands functional augmentation to concurrently incorporate antibacterial, immunomodulatory, ROS‐regulatory, and osteogenic capacities. Here, we designed the PC‐OGP@TOB capsule by leveraging the amino‐rich domains of K6‐OGP and TOB. Controlled dual loading was achieved via Schiff base reactions between quinones generated from the oxidation of phenolic hydroxyl groups in PC capsules and the amine groups of TOB/K6‐OGP (**Scheme** **1**A). In *Staphylococcus aureus* (*S. aureus*) infected osteomyelitis models (Scheme 1B), PC‐OGP@TOB exhibited: (1) Bactericidal efficacy via dynamic TOB capture‐release, (2) Immunomodulatory activity by polarizing macrophages toward the M2 phenotype and inducing angiogenesis, and (3) The capsule promotes bone regeneration by synergistically upregulating collagen II and osteocalcin (OCN) to remodel the ECM microenvironment (Scheme 1C). Furthermore, it activates the AMPK pathway to enhance mitochondrial energy metabolism in osteoblasts and augments PI3K‐Akt pathway activity (mediated by RUNX2/BMP2), driving an osteoblast proliferation‐differentiation cascade, thereby achieving multidimensional regulation of bone regeneration. We believe that the multifaceted therapeutic approach presented in this study offers a new direction for the treatment of osteomyelitis.

## 2 Results and Discussion

### 2.1 Preparation of PC‐O@TOB Capsules

**Figure 1:**
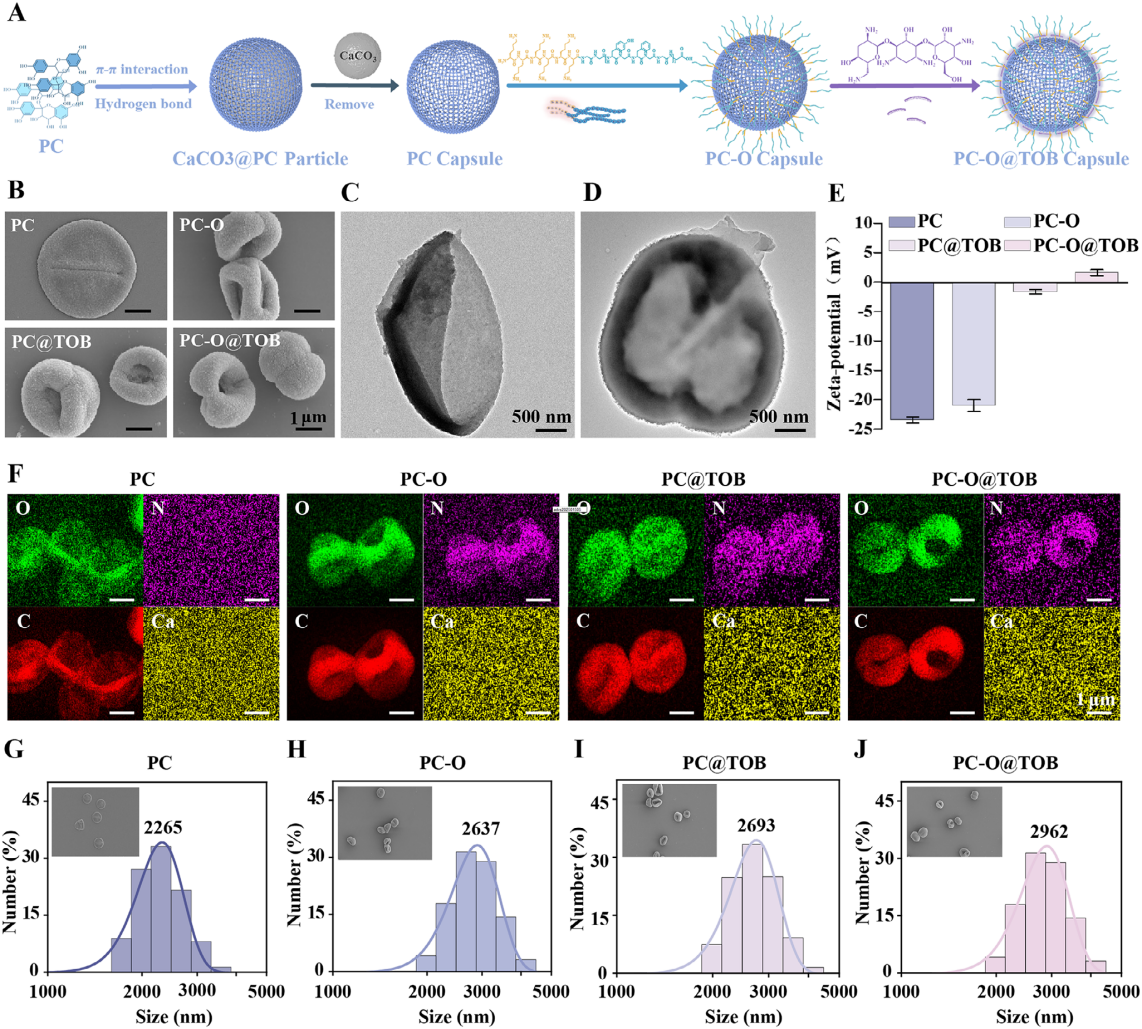
Preparation and characterization of PC‐O@TOB capsules. A) Schematic illustration of the fabrication process for PC‐O@TOB capsules. B) SEM images of PC capsules, oxidized procyanidin (PC‐O) capsules, PC@TOB capsules, and PC‐O@TOB capsules. C) TEM image of PC capsules. D) TEM image of PC‐O@TOB capsules. E) Zeta potential analysis of PC capsules, PC‐O capsules, PC@TOB capsules, and PC‐O@TOB capsules (N═4). F) EDS spectra of PC capsules, PC‐O capsules, PC@TOB capsules, and PC‐O@TOB capsules from SEM analysis, presented from left to right. G‐J) The size distribution profiles of PC capsules, PC‐O capsules, PC@TOB capsules, and PC‐O@TOB capsules, respectively.

The coprecipitation method was utilized to incorporate PC into calcium carbonate (CaCO₃) templates, as previously reported.^[^ ^28^, ^29^ ^]^ After removing the template with hydrochloric acid, hollow PC capsules were fabricated by employing the non‐covalent forces and π‐π stacking among PC solution.^[^ ^30^ ^]^ By adjusting the pH value to 8.0, the functional polypeptide K6‐OGP and TOB were sequentially conjugated onto PC capsules to produce PC‐O and PC‐O@TOB capsules, respectively (**Figure** **1**A). When the pH is adjusted to 10.0, the solubility of PC surges, causing the disintegration of PC capsules. Conversely, at a pH of 6.0, the amino groups of TOB in the Schiff base reaction are protonated, reducing their nucleophilicity. This impedes the formation of Schiff base bonds with the carbonyl groups generated from the oxidation of phenolic hydroxyl groups in PC, hindering component conjugation and decreasing reaction efficiency (Figure S1, Supporting Information).

Scanning electron microscopy (SEM) images illustrated a marked increase in capsule thickness and structural complexity post‐loading with K6‐OGP and TOB, confirming effective functionalization of the PC capsules (Figure 1B). Transmission electron microscope (TEM) analysis revealed the morphological difference between PC capsules and PC‐O@TOB capsules, with the latter exhibiting an obvious external coating layer (Figure 1C‐D). When TAMRA‐K6‐OGP (a fluorescently tagged variant of K6‐OGP) was conjugated to PC capsules, intense red fluorescence signaled successful polypeptide loading (Figure S2, Supporting Information). Notably, subsequent TOB loading produced no discernible reduction in fluorescence intensity, implying that TOB incorporation does not competitively displace surface‐bound K6‐OGP. The measurement results of zeta potential in Figure 1E further supported the successful loading of K6‐OGP and TOB. The surface of the initial PC capsule had a negative charge of ≈ ‐23 mV. After loading K6‐OGP and TOB, the zeta potential changed to +1.6 mV. This step‐change could be attributed to the multiple lysine residues in K6‐OGP and the multiple amino groups on TOB, which significantly regulated the surface charge of the capsules (Figure 1E). To further confirm the presence of functional elements in the capsules, energy dispersive spectroscopy (EDS) analysis was conducted (Figure 1F). The results showed that nitrogen element (N) was detected in PC‐O, PC@TOB, and PC‐O@TOB capsules, indicating the successful loading of polypeptides and antibiotics. The content of calcium element (Ca) was negligible, demonstrating the success of removing CaCO₃ with hydrochloric acid. Dynamic light scattering (DLS) profiles revealed a substantial capsules size increase from 2265 to 2960 nm, consistent with the incorporation of K6‐OGP and TOB (Figure 1G). These data collectively indicated that the K6‐OGP and TOB had been successfully loaded into the capsules and confirmed the structural stability of the capsules after functionalization.

### 2.2 Formation Mechanism and Characterization of PC‐O@TOB Capsules

**Figure 2:**
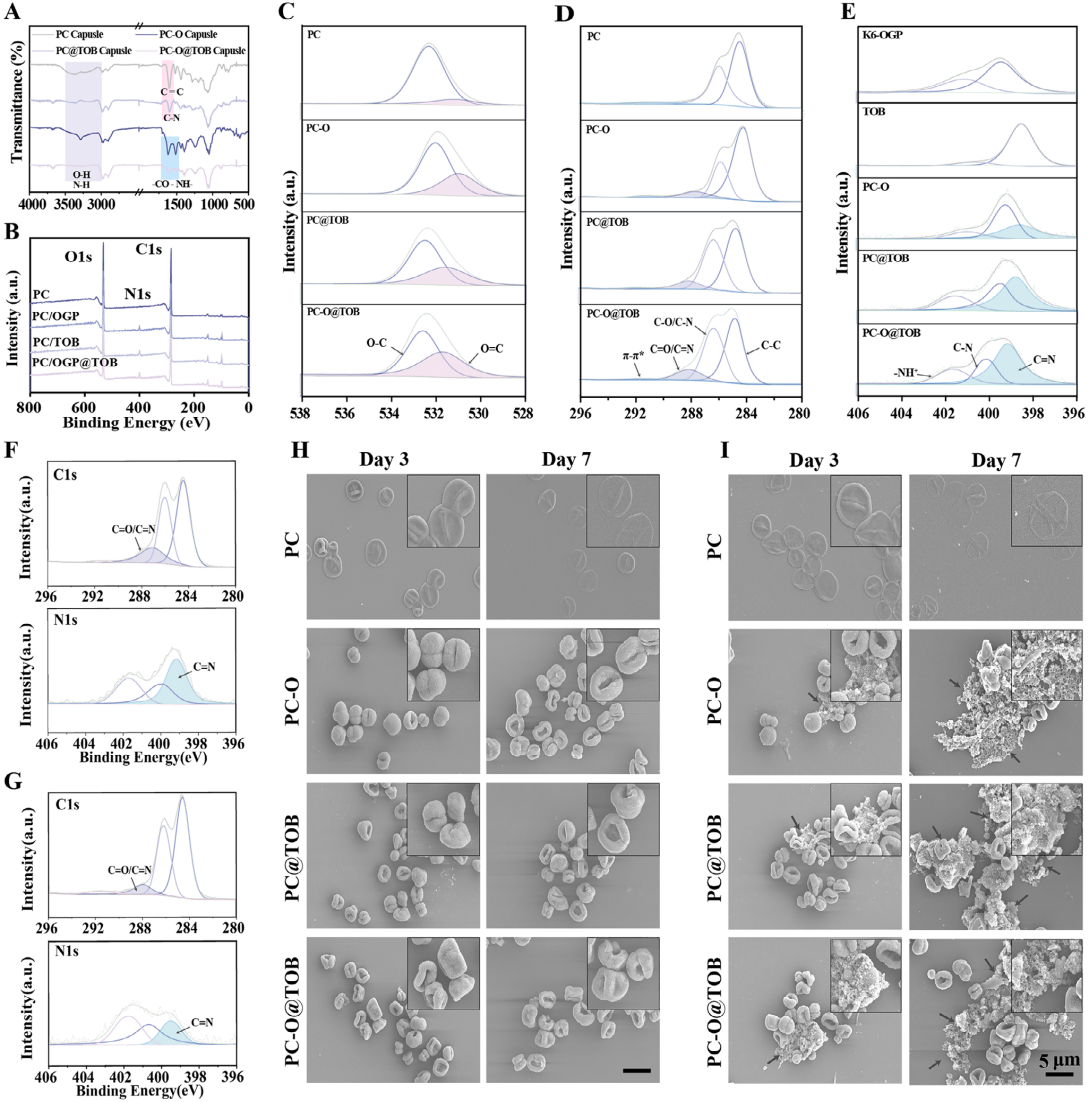
Formation and characterization of capsules. A) FTIR mapping of PC, PC‐O, PC@TOB, and PC‐O@TOB capsules. B) XPS spectra of PC, PC‐O, PC@TOB, and PC‐O@TOB capsules. C─E) Peak deconvolution analysis of O 1s, C 1s, and N 1s for PC, PC‐O, PC@TOB, and PC‐O@TOB capsules, including K6‐OGP and TOB. F) XPS analysis of PC‐O@TOB capsules after 3 days at pH 7.0 and G) at pH 5.0. H) SEM images of PC, PC‐O, PC@TOB, and PC‐O@TOB capsules following 3 and 7 days of incubation at pH 7.0 and I) at pH 5.0.

Fourier transform infrared spectroscopy (FTIR) analysis exhibited a broad O─H stretching vibration peak within the range of 3500‐3000 cm⁻¹ and a benzene ring C═C stretching vibration peak within the range of 1700‐1500 cm⁻¹ for PC. TOB presented both N─H and O─H stretching vibration peaks in the range of 3500‐3000 cm⁻¹ and a C─N stretching vibration peak in the range of 1700‐1500 cm⁻¹. K6‐OGP showed a lysine residue amino N─H stretching vibration peak in the range of 3500‐3000 cm⁻¹ and a peptide bond (─CO─NH─) stretching vibration peak in the range of 1700‐1500 cm⁻¹ (Figure S3, Supporting Information). Notably, the FTIR peaks of PC capsules in the 3500‐3000 cm⁻¹ and 1700‐1500 cm⁻¹ regions closely matched those of pure PC, indicating PC dominated composition. For PC@TOB capsules, the presence of TOB‐specific functional groups (C─N) altered absorption peaks observed at 1700‐1500 cm⁻¹, confirming successful TOB loading and its interaction with PC. Additionally, both PC‐O and PC‐O@TOB capsules exhibited peptide bond (─CO─NH─) absorption peaks at 1700‐1500 cm⁻¹, validating K6‐OGP incorporation. The complex peak profile of PC‐O@TOB capsules suggested multicomponent interactions (**Figure** **2**A).

X‐ray photoelectron spectroscopy (XPS) further analyzed the surface chemical environment of PC, PC‐O, and PC‐O@TOB capsules (Figure 2B). After loading K6‐OGP and TOB, the binding energies in the C_1s_ and O_1s_ regions were significantly changed, reflecting the reconstruction of the surface chemical environment of the capsules. At the same time, an obvious N_1s_ peak appeared in the XPS spectrum, further confirming the successful loading of K6‐OGP and TOB, and the content of N element significantly increased to 4.64% (Figure S4A, Supporting Information). To deeply analyze the action mechanism of PC‐O@TOB capsules, we performed peak separation analysis on O, C, and N elements. The O 1s peak separation results showed that the O═C peak was significant in PC‐O, PC@TOB, and PC‐O@TOB capsules, indicating that in a weakly alkaline environment, PC was easily oxidized to generate quinones and combine with amino groups through Schiff base reactions to achieve efficient loading (Figure 2C). The C 1s peak separation analysis showed that the area of the C═O/C═N peak in PC‐O, PC@TOB, and PC‐O@TOB capsules significantly increases (Figure 2D). In addition, the π‐π interaction peak at 292 cm⁻¹ further confirmed that the formation of PC capsules was closely related to π‐π interaction. Compared with PC capsules, PC‐O, PC@TOB, and PC‐O@TOB capsules all showed significant C═N peaks, indicating that K6‐OGP and TOB were loaded through Schiff base interaction (Figure 2E). During the aqueous phase mixing reaction, the polyphenol part in PC capsules was oxidized, and the generated quinone intermediate reacted with the polylysine in K6‐OGP and multiple amino groups in TOB through Michael addition and Schiff base reactions.^[^ ^29^, ^31^, ^32^ ^]^ At the same time, to prove the universality of our method, we loaded two other aminoglycoside antibiotics, gentamicin (GM) and kanamycin (KM) respectively, onto PC capsules. As observed under SEM (Figure S5, Supporting Information), it could be seen that the capsule structure became fuller and more 3D, indicating that the capsules could achieve effective loading of aminoglycoside antibiotics.

To verify the acidic disintegration properties of the capsules, they were incubated in phosphate buffer solutions (pH 7 and 5). XPS analysis of PC‐O@TOB capsules was performed on the third day under both pH conditions, while SEM observations were conducted on days 3 and 7 to assess morphological changes. At pH 7, XPS results revealed a nitrogen content of 7.36%, along with C ═ O/C ═ N and C = N peak areas of 20.6% and 43.6%, respectively (Figure 2F). In contrast, at pH 5, the nitrogen content dropped significantly to 2.85%, accompanied by reductions in C = O/C = N and C = N peak areas to 7.55% and 21.6%, respectively (Figure 2G). Consistent with these findings, SEM imaging showed stable capsule morphology at pH 7 (Figure 2H), whereas PC‐O, PC@TOB, and PC‐O@TOB capsules disintegrated to varying degrees on the third and seventh days at pH 5, confirming the responsive disintegration properties of the Schiff base‐modified capsules in an acidic environment (Figure 2I). Furthermore, we placed the PC‐O@TOB capsules in water, PBS, and DMEM environments for one week and then analyzed their surface structures under SEM to verify their stability. It was found that the structure remained basically unchanged (Figure S6, Supporting Information), indicating that the capsules had good stability. The schematic diagram of polyphenol‐based scavenging of ROS was shown in Figure S7A (Supporting Information). To evaluate the antioxidant capacity of the capsules, we detected the antioxidant properties of the capsules through DPPH and ABTS antioxidant kits (Figure S7B‐D, Supporting Information). The results showed that although some PC may be oxidized during the loading process of K6‐OGP and TOB, most of the PC remains unoxidized. Therefore, the antioxidant capacities of PC capsules, PC‐O, PC@TOB, and PC‐O@TOB capsules remain basically the same. Our research indicated that the PC‐O@TOB capsules not only have efficient drug loading capacity but also responsively disintegrate in an acidic environment for controlled controllable drug release. In addition, although some PC was oxidized during the loading process, the antioxidant capacity of the capsules was not affected, demonstrating its adaptability and functional durability in complex biological environments.

### 2.3 Biocompatibility and Osteogenesis Promotion of Capsules In Vitro

**Figure 3:**
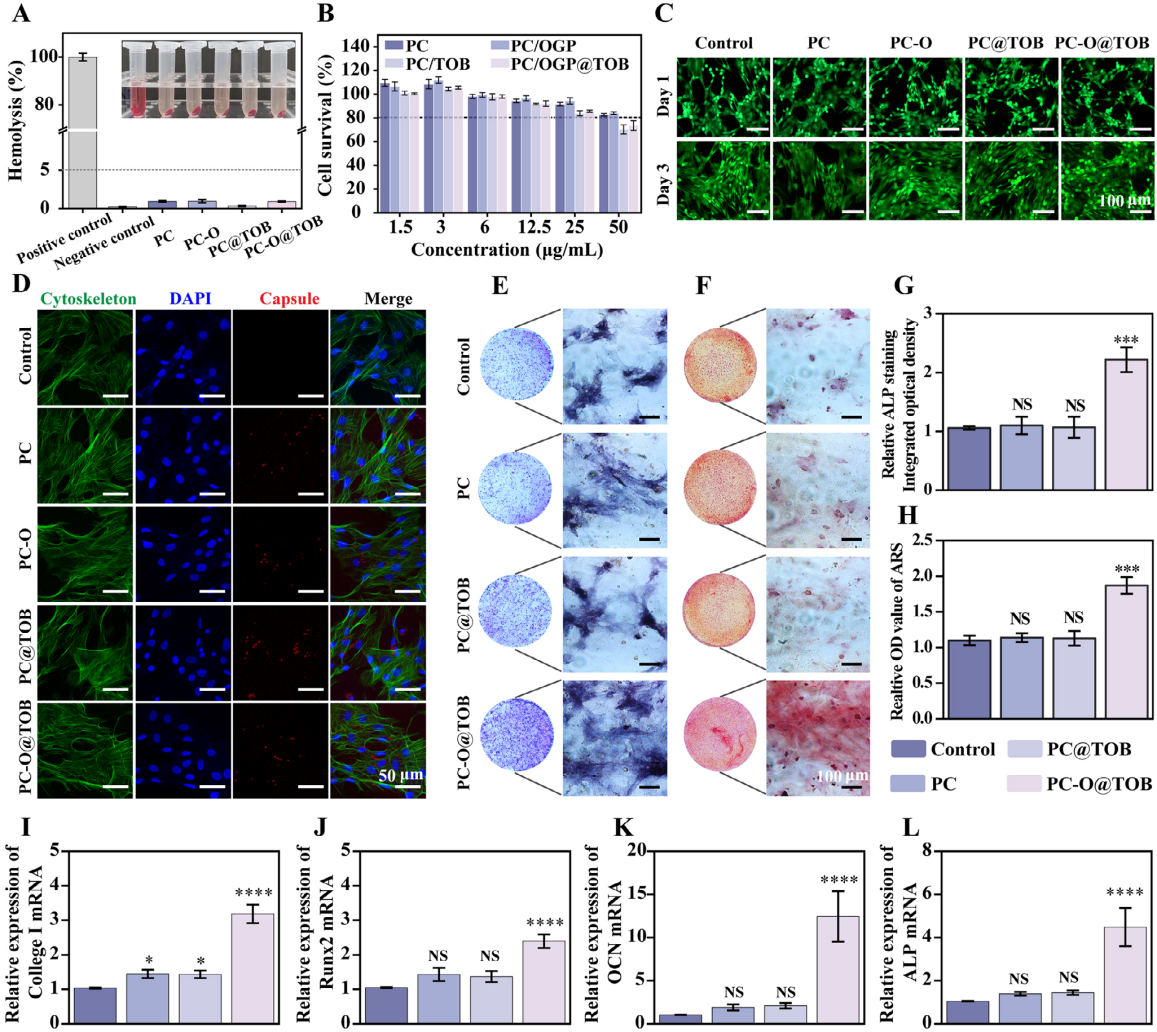
In vitro evaluation of capsules biocompatibility and osteogenic activity. A) Hemocompatibility of different capsules, with corresponding images displayed in the upper right corner of each group (N = 4). B) Viability of BMSCs cell after 3 days of incubation with capsules (N = 4). C) CLSM images of BMSCs stained with calcein‐AM (live cells, green) and propidium iodide (PI; dead cells, red) following 1‐ and 3‐day incubation with capsules. D) BMSC morphology post‐capsule internalization. Cytoskeleton: FITC‐phalloidin (green); nuclei: DAPI (blue); capsule autofluorescence (red). E) ALP staining of BMSCs cultured in osteogenic medium supplemented with different samples on day 14. F) ARS staining of BMSCs cultured in osteogenic medium supplemented with different samples on day 21. The quantitative results of G) ALP staining and H) ARS staining (N = 3). qPCR analysis of osteogenesis‐related gene expression in BMSCs after 14 days of osteogenic induction. The genes analyzed included I) collagen I, J) Runx2, K) OCN, and L) ALP (N = 3).

The biocompatibility and osteogenic functionality of the capsules were evaluated. First, the hemolysis rates of PC, PC‐O, PC@TOB, and PC‐O@TOB capsules were <5% (**Figure** **3**A), indicating no significant hemolytic activity upon blood exposure, affirming their hemocompatibility for in vivo use. The viability of bone marrow mesenchymal stem cells (BMSCs) cultured with the capsules for 3 days was assessed by the CCK‐8 assay. It was found that the cell viability was all above 80% at a concentration of 25 µg mL^−1^ (Figure 3B). Live‐dead staining with inverted fluorescence microscopy confirmed high BMSC viability across all groups after 1 and 3 days (Figure 3C). Morphological analysis via confocal laser scanning microscopy (CLSM) revealed efficient capsule internalization by BMSCs. The internalized capsules would degrade and release in the lysosomes while preserving cytoskeletal integrity (Figure S8, Supporting Information), further validating the structural biocompatibility of TOB/K6‐OGP co‐loaded capsules (Figure 3D).

In the bone repair process, the main cells responsible for bone formation are BMSCs, which can differentiate into osteoblasts.^[^ ^33^, ^34^ ^]^ K6‐OGP,^[^ ^32^, ^37^ ^]^ which incorporates OGP, enhances osteoblast proliferation and differentiation, thereby accelerating bone regeneration.^[^ ^35^, ^36^ ^]^ The alkaline phosphatase (ALP) staining results showed that the osteoblast activity in the PC‐O@TOB group was significantly enhanced on day 7 and 14 (Figure 3E; Figure S9, Supporting Information). The PC‐O@TOB group also showed the advantage in mineralization in alizarin red S (ARS) staining, especially reaching the highest mineralization degree on day 7 and 21 (Figure 3F; Figure S9, Supporting Information). Quantitative analysis confirmed the highest ALP activity and mineralization levels in this group (Figure 3G,H). These results indicated that the PC‐O@TOB capsule not only significantly increased the activity of osteoblasts but also accelerated the bone mineralization process, possibly acting by regulating osteogenesis‐related signaling pathways through K6‐OGP. The results of real‐time quantitative PCR (qPCR) analysis further supported this conclusion. The expression levels of osteogenesis‐related genes, including collagen type I (Col I), Runx2, OCN, and ALP, were detected by RT‐qPCR on the 14th day of osteogenic induction, The results showed that the PC‐O@TOB group significantly upregulated the expression of key genes related to bone formation (Figure 3I‐L). The upregulation of these genes not only indicated that this capsule could effectively promote the differentiation and mineralization of bone cells but also implied its great potential in bone tissue regeneration.

### 2.4 Capsule‐Mediated ROS Scavenging and Modulation of Macrophage Polarization

**Figure 4:**
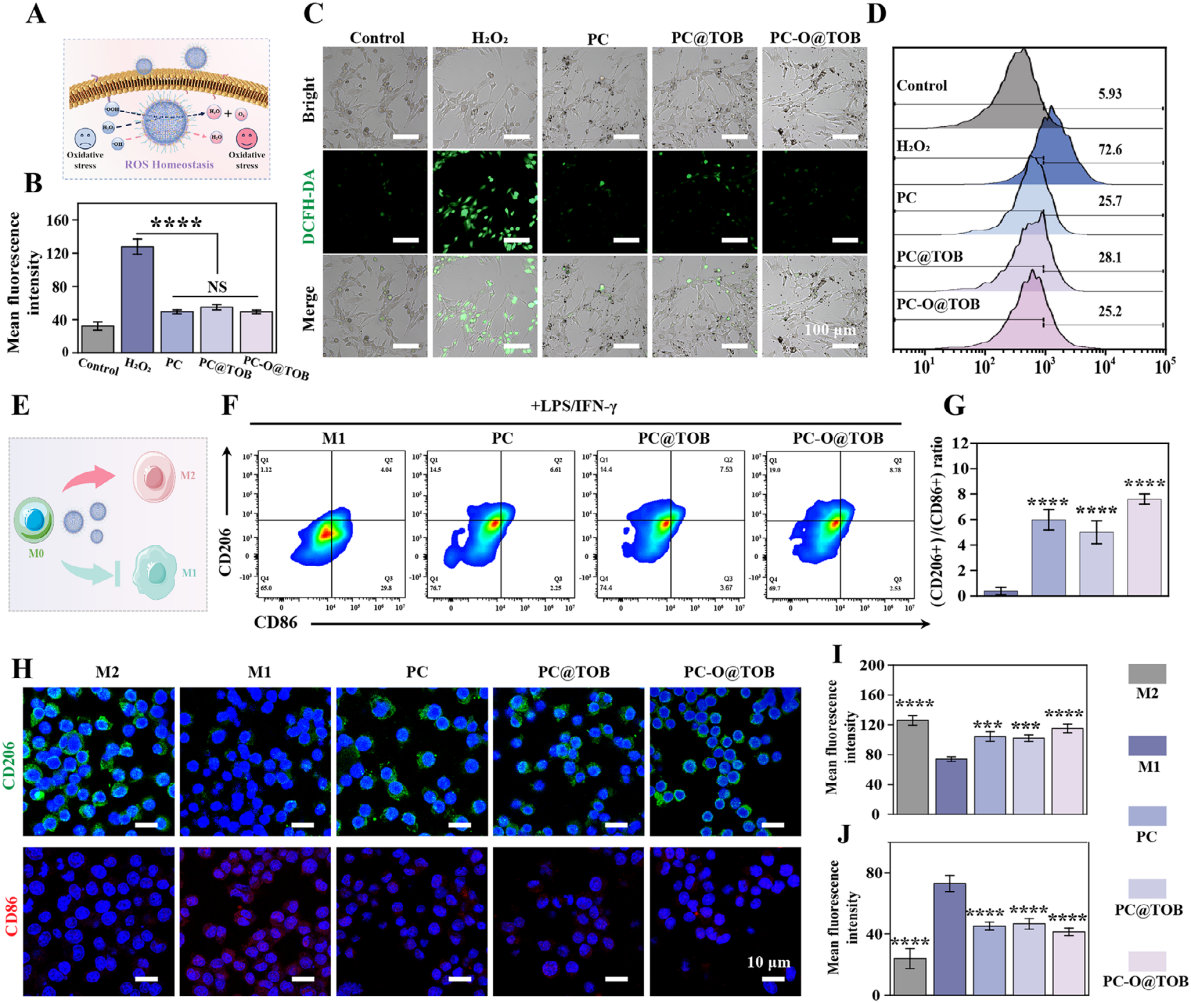
Capsule‐mediated ROS scavenging and macrophage polarization modulation. A) Schematic illustrating the ROS‐scavenging mechanism of the capsules. B) Quantitative analysis of intracellular ROS levels using DCFH‐DA. C) Fluorescence microscopy images visualizing ROS levels via DCFH‐DA (green); images correlate with the quantitative data in (B). D) Flow cytometric quantification of intracellular ROS levels using DCFH‐DA fluorescence intensity. E) Schematic depicting capsule‐mediated regulation of RAW 264.7 macrophage polarization. F) Flow cytometry analysis of M1 (CD86⁺) and M2 (CD206⁺) macrophage surface marker expression. G) Statistical analysis of the flow cytometry results in (F)(N = 3). H) Immunofluorescence staining of M1 (CD86, red) and M2 (CD206, green) macrophage markers. Quantitative analysis of I) CD206⁺ and J) CD86⁺ from (H) (N = 3).

Osteomyelitis is a complex infectious disease characterized by abnormally elevated levels ofROS and immune imbalance.^[^ ^12^, ^38^ ^]^ Persistent ROS accumulation not only induces oxidative stress, exacerbating tissue damage, but also impedes bone regeneration.^[^ ^39^, ^40^ ^]^ Consequently, restoring macrophage polarization balance—particularly promoting transition from pro‐inflammatory M1 to reparative M2 phenotypes—coupled with effective ROS scavenging, constitutes a critical therapeutic strategy for osteomyelitis. PC, PC‐O, PC@TOB and PC‐O@TOB capsules could effectively scavenge excessive ROS, eliminating superoxide (·O₂⁻), hydrogen peroxide (H₂O₂), and hydroxyl radicals (·OH) via the antioxidant activity of their PC components, converting these species into water (H₂O) and oxygen (O₂) (**Figure** **4**A). This scavenging mechanism reduced the cellular damage caused by oxidative stress and decreased the accumulation of ROS in the inflammatory microenvironment of osteomyelitis. 2′,7′‐dichlorofluorescein diacetate (DCFH‐DA) was a fluorescent probe used to quantitatively detect the intracellular ROS level. Fluorescence microscope images showed that the ROS level in all capsule treatment groups was significantly reduced, proving the antioxidant potential of the capsules in the in vivo environment (Figure 4B,C). Flow cytometry analysis also supported this conclusion (Figure 4D). Compared with the H₂O₂ treatment group (with a ROS positive rate of 72.6%), the ROS positive rates of all capsule treatment groups were reduced to around 25%, confirming the significant ROS scavenging effect of the PC, PC@TOB, and PC‐O@TOB groups.

Macrophage polarization modulation is pivotal for osteomyelitis resolution due to its central role in balancing pro‐inflammatory (M1) and anti‐inflammatory/reparative (M2) phenotypes. Schematic representation of Mouse Mononuclear Macrophages cells (RAW 264.7) macrophage regulation by capsules is shown in Figure 4E. Lipopolysaccharide (LPS) and interferon‐γ (INF‐γ) induced macrophage polarization, whereas capsule treatments suppressed M1 polarization (CD86⁺) and enhanced M2 polarization (CD206⁺). The flow cytometry results in Figure 4F,G showed that all capsule‐treated groups significantly reduced the proportion of M1 macrophages from 29.8% to ≈2.5%, and substantially increased the proportion of M2 macrophages, especially in the PC‐O@TOB group, where the proportion of M2 macrophages was increased from 1.1% to 19%. The ability of the PC‐O@TOB group to promote macrophage polarization was attributed to the anti‐inflammatory and immunomodulatory functions of PC. On the other hand, the PC‐O@TOB group showed better effect, which may be related to the presence of OGP. As a polypeptide with osteogenic and anti‐inflammatory properties, it could actively promote the macrophage polarization process by regulating signal pathways and sending healing‐promoting signals. Specifically, K6‐OGP not only promoted the differentiation of osteoblasts but also alleviated inflammation by inducing the production of M2 macrophages. The immunofluorescence staining in Figure 4H further demonstrated that compared with the positive control group, the green fluorescence intensity increased and the red fluorescence weakened in all capsule groups, indicating that the proportion of M2 macrophages was significantly increased and the M1 macrophages were decreased in the capsule treatment group. Especially in the PC‐O@TOB treated group, the expression of CD206, marked by green fluorescence, was more intense, indicating a significant increase in the proportion of M2 macrophages (Figure 4I,J). It was basically consistent with the trend of flow cytometry results. In summary, PC‐O@TOB jointly promoted the repair of damaged tissues and the treatment effect of osteomyelitis by regulating macrophage polarization to reduce inflammation, scavenging ROS to reduce oxidative stress, and overall controlling the inflammatory response.

### 2.5 In Vitro Regulation of Osteogenic Differentiation and Angiogenesis Through Macrophage Polarization

In osteomyelitis treatment, polarized M2 macrophages play a pivotal role.^[^ ^12^, ^41^ ^]^ These cells mitigate inflammation and enhance tissue repair by secreting anti‐inflammatory cytokines such as interleukin‐10 (IL‐10) and transforming growth factor‐β (TGF‐β).^[^ ^42^ ^]^ M2 macrophages not only suppress inflammation but also drive bone regeneration by upregulating osteogenic mediators like bone morphogenetic protein‐2 (BMP‐2).^[^ ^43^, ^44^ ^]^ Additionally, M2 macrophage‐derived angiogenic factors, such as vascular endothelial growth factor (VEGF), facilitate neovascularization and tissue healing.^[^ ^45^, ^46^ ^]^ Their dominance during the late stages of bone repair counteracts the early pro‐inflammatory milieu induced by M1 macrophages, enabling tissue remodeling and repair. Thus, M2 polarization is critical for controlling osteomyelitis progression and restoring bone integrity.

**Figure 5:**
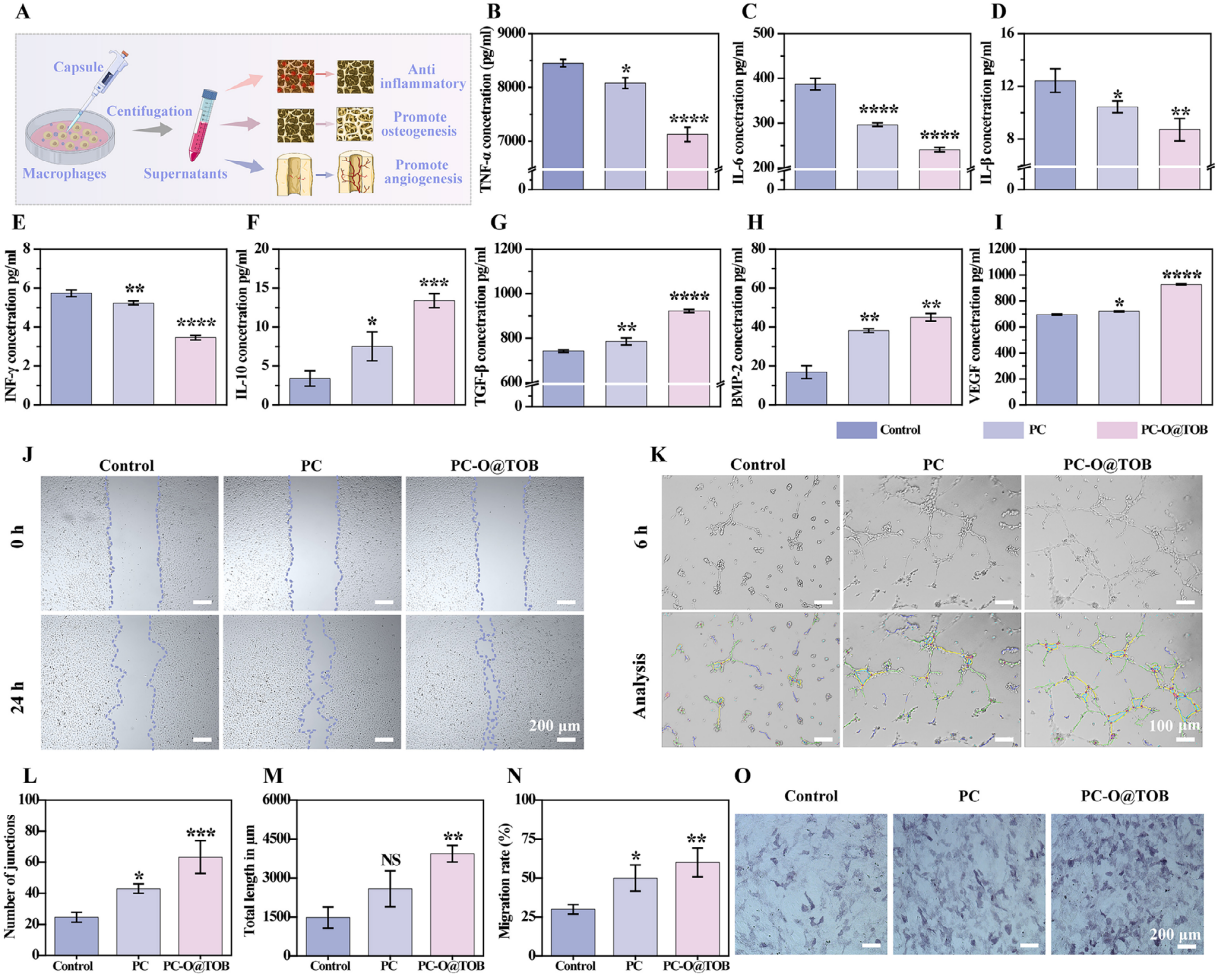
In vitro regulation of osteogenic differentiation and angiogenesis through macrophage polarization. A) Schematic illustration of the macrophage polarization‐mediated mechanistic regulation of osteogenesis and angiogenesis. B–I) ELISA quantification of cytokines in macrophage culture supernatants across experimental groups: (B) pro‐inflammatory TNF‐α, (C) IL‐6, (D) IL‐1β, (E) IFN‐γ, (F) anti‐inflammatory IL‐10, (G) TGF‐β, (H) osteogenic BMP‐2, and (I) angiogenic VEGF (N = 3). J) HUVEC migration after 24 h incubation with conditioned media from treated RAW 264.7 macrophages, assessed via scrape‐wound (scratch) assay. K) In vitro angiogenesis assay evaluating HUVEC tube formation after 6 h exposure to macrophage‐conditioned media. L) Quantification of branch points (network complexity) and M) total capillary length from panel (K) (N = 3). N) Migration distance quantification of HUVECs from panel (J) (N = 3). O)ALP staining of BMMSCs cultured for 7 days in osteogenic differentiation medium supplemented with macrophage‐conditioned media, indicating osteogenic differentiation potential.

To analyze the expression of cytokines after capsule‐regulated polarization of macrophage RAW 264.7 cells, we collected the culture supernatants of capsule‐treated cells three days later and detected the protein levels of several typical inflammation, osteogenesis, angiogenesis, and related cytokines by enzyme‐linked immunosorbent assay (ELISA) (**Figure** **5**A). In osteomyelitis, elevated pro‐inflammatory cytokines (TNF‐α, interleukin‐6 (IL‐6), interleukin‐1β (IL‐1β), IFN‐γ) exacerbate tissue damage and inflammation. Capsule treatment, particularly PC‐O@TOB, markedly suppressed these factors, demonstrating anti‐inflammatory efficacy (Figure 5B‐E). It may be attributed to the fact that the capsules effectively inhibited the activation of M1‐type macrophages and slowed down the inflammatory response, thereby reducing tissue damage. In addition, the PC‐O@TOB capsule significantly upregulated the expression of IL‐10 (Figure 5F). BMP‐2, a key osteogenic inducer, and TGF‐β, which mediates anti‐inflammatory and chondro‐osseous repair processes, were elevated in the PC‐O@TOB group, suggesting dual roles in inflammation resolution and bone regeneration (Figure 5G‐H). The enhancement of VEGF in the PC‐O@TOB group apparently promoted the formation of new blood vessels and improved the blood supply in the damaged area, thus accelerating the tissue repair and regeneration (Figure 5I).

The ability of PC‐O@TOB capsules to promote angiogenesis was further confirmed by culturing human umbilical vein endothelial cells (HUVECs) with RAW 264.7 cell culture supernatant treated with PC‐O@TOB capsules. The results of the scratch assay revealed that the RAW 264.7 cell supernatant treated with the capsule group promoted cell migration, while the effect was more pronounced in the PC‐O@TOB group (Figure 5J,N). In addition, the tube formation showed that compared with the control group and the PC group, the number of connections and tube length of HUVECs in the PC‐O@TOB group were significantly increased, which could better promote angiogenesis (Figure 5K‐M). Meanwhile, we also found that during the osteogenesis induced by RAW 264.7 cell supernatant, the ALP staining results revealed more positive areas in the PC‐O@TOB group (Figure 5O), indicating that PC‐O@TOB could better promote osteogenesis.

### 2.6 Bacterial Capture and Antibacterial Activity of Capsules In Vitro

**Figure 6:**
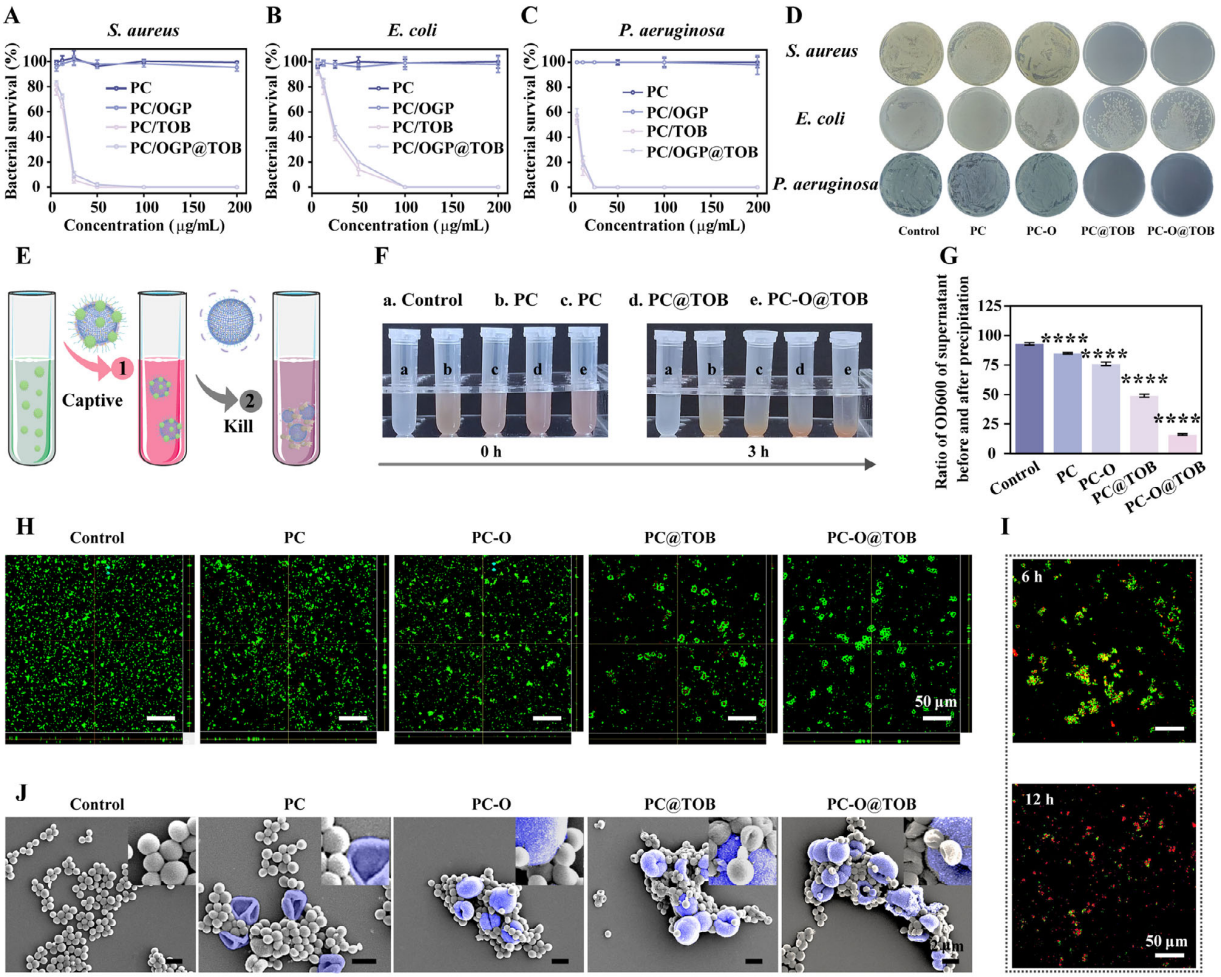
In vitro bacterial capture and antibacterial activity of capsules. A‐C) Bacterial survival rates of *S. aureus* (A), *E. coli* (B), and *P. aeruginosa* (C) after 24‐hour treatment with different capsules (N = 3). D) CFU analysis of bacterial viability on agar plates after treatment with different capsules at 25 µg mL^−1^. E) Schematic illustration of bacterial capture by capsules. F) Sedimentation of *S. aureus* after co‐incubation with different capsules for 0 and 3 h. G) Turbidity analysis of the supernatant from the 3‐hour incubation in (F) (N = 3). H) CLSM images of *S. aureus* after 3 h of co‐incubation with different capsules. Live bacteria are stained green. I) CLSM images of *S. aureus* after 6 and 12 h of co‐incubation with different capsules, using live/dead bacterial staining; live bacteria are stained green, while dead bacteria are stained red. J) SEM images of *S. aureus* after 12 h of co‐incubation with different capsules (N = 4).

*S. aureus* is a predominant osteomyelitis pathogen, notably in long bones like the tibia due to their enclosed structure and limited vascularity, which facilitates bacterial persistence and chronic inflammation. Free‐floating bacteria in the tibial marrow cavity often evade conventional antibiotics, necessitating strategies for simultaneous capture and eradication. As shown in **Figure** **6**A–C, after treatment with PC@TOB and PC‐O@TOB capsules for 24 h, the survival rates of *S. aureus* and Pseudomonas aeruginosa (*P. aeruginosa*) at 25 µg mL^−1^ concentration were nearly 0, and the antibacterial effect was almost 100% while the survival rate of Escherichia coli (*E. coli*) was maintained at about 40%, which indicated that the capsules exhibited significant broad‐spectrum antibacterial activity, especially in targeting *S. aureus* and *P. aeruginosa*. The antimicrobial properties of the capsules were further demonstrated by the colony forming unit (CFU) analysis (Figure 6D). Notably, the antibacterial effect of PC‐O@TOB capsules was almost the same as that of PC@TOB capsules, suggesting the similarity in drug release and bacterial capture mechanisms between the two. Other capsule groups (PC and PC‐O groups) did not show significant antibacterial ability, indicating that TOB played a key role in the antibacterial mechanism. We additionally fabricated aminoglycoside‐loaded capsules (PC@GM and PC@KM) and evaluated their antibacterial activity via inhibition zone assays. Dose‐responsive inhibition zones (0.25–1 mg mL^−1^) observed for PC@GM, PC@KM, and PC@TOB (Figure S10, Supporting Information) corroborated their concentration‐dependent antibacterial efficacy, demonstrating the broad applicability of our PC capsule platform for targeted delivery of diverse aminoglycoside antibiotics.

The phenol hydroxyl‐rich PC drives adhesion through hydrogen bonding and van der Waals interactions with bacterial surface‐associated proteins. Surface charge conversion via TOB‐mediated enhances electrostatic attraction to negatively charged bacterial membranes. Aminoglycoside‐specific interactions with surface polysaccharides further enhance capture efficiency. As depicted in Figure 6E, after initial bacterial immobilization by PC‐O@TOB capsules, localized TOB release eradicates the captured bacteria, achieving dual‐function integration of capture and targeted bactericidal activity. As shown in Figure 6F, when different types of capsules were mixed with the same concentration of *S. aureus* for 0 h and 3 h, it could be seen that the supernatants of PC@TOB and PC‐O@TOB were clearer compared to the control group, indicating that the bacteria had been captured and precipitated by the capsules. The OD value analysis further revealed the difference in bacterial capture capabilities of different capsules (Figure 6G). After co‐incubation with *S. aureus* for 3 h, the OD values of the supernatants of all capsule groups were significantly reduced, indicating that the bacteria were effectively captured by the capsules. Among them, the PC@TOB and PC‐O@TOB groups showed the largest decrease of the OD values, suggesting that TOB modification enhanced the capture ability.

The CLSM images showed that after co‐incubation with *S. aureus* for 3 h, a large number of green bacteria in the PC@TOB and PC‐O@TOB capsules groups were aggregated on the capsule surface, indicating that the bacteria were significantly captured (Figure 6H; Figure S11A, Supporting Information). Live/dead bacterial staining results revealed that after co‐culturing the capsules with *S. aureus* for 6 h, some of the bacteria changed from green to red, indicating that some bacteria had died; by 12 h, almost all bacteria of the bacteria had been killed and showed red staining (Figure 6I). The SEM images further showed the morphological changes of *S. aureus* after co‐incubation with the capsules for 12 h (Figure 6J). The PC‐O@TOB group, the bacteria were tightly wrapped around the capsule surface with obvious shrinkage and leakage of cellular contents, suggesting that the bacteria had been severely destroyed by TOB. This result was consistent with the live/dead staining result, further confirming the significant antibacterial effect of the PC‐O@TOB capsule. When pathogens such as bacteria invade the bone marrow cavity, bone tissue, and surrounding soft tissues, they may form biofilms locally. We constructed biofilms in vitro using *S. aureus* and found that both PC@TOB and PC – O@TOB capsules exhibited powerful biofilm elimination capabilities and were able to effectively remove a large amount of biofilm (Figure S11B,C, Supporting Information). The dual‐action capture‐kill mechanism of PC‐O@TOB capsules showcases significant therapeutic potential for the management of osteomyelitis, particularly in anatomically restricted compartments like the tibia.

### 2.7 Treatment of Osteomyelitis in an Animal Model Using Capsules

**Figure 7:**
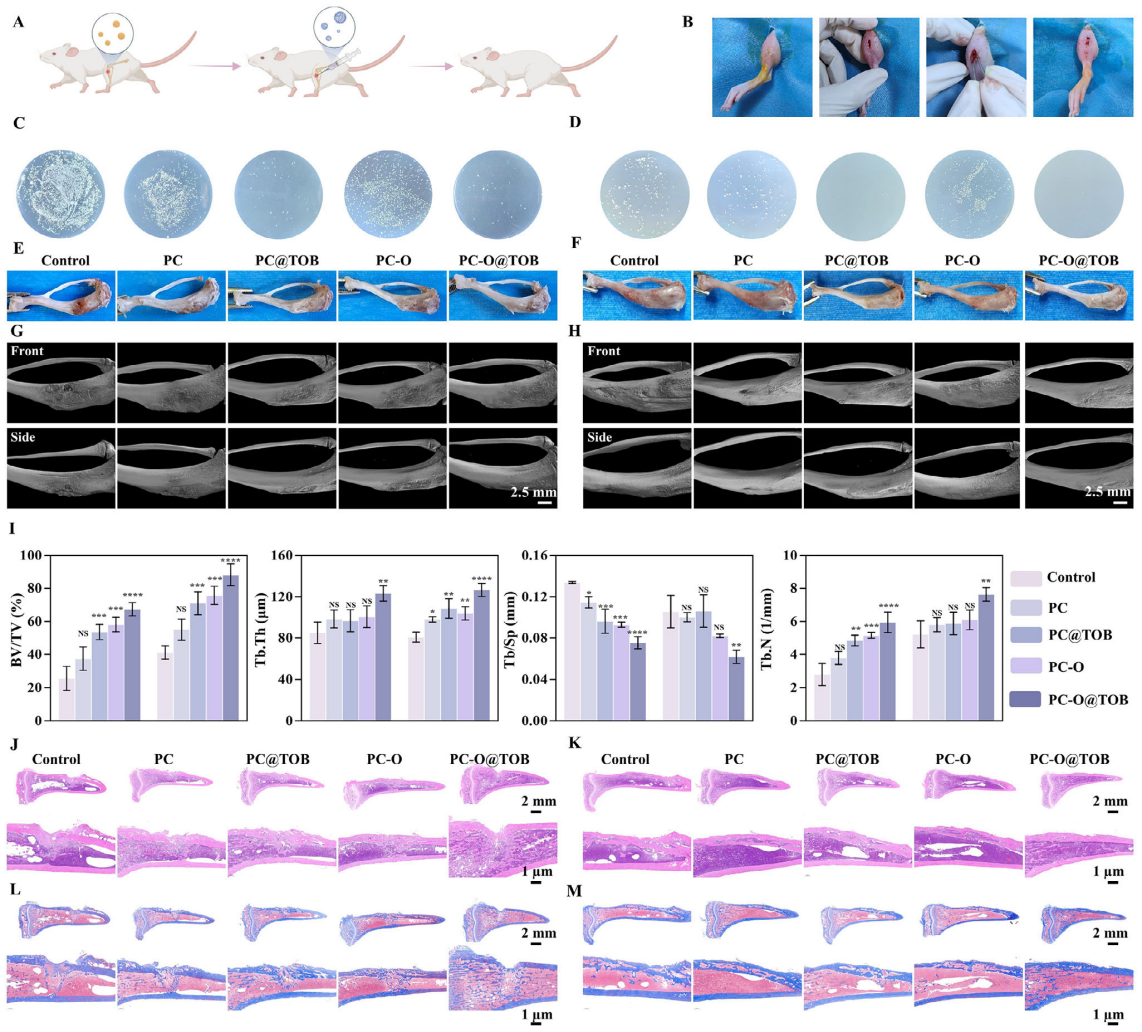
Treatment of osteomyelitis in an animal model using capsules. A) Schematic representation of capsule‐based osteomyelitis treatment. B) Workflow of osteomyelitis induction. C) CFU results from PBS flushes of the infected area of the tibia 15 days post‐treatment with different capsules. D) CFU results from PBS flushes of the infected area of the tibia 30 days post‐treatment with different capsules. E) Images of the infected bone site after 15 days of treatment with different capsules. F) Images of the infected bone site after 30 days of treatment with different capsules. G) 3D micro‐CT images of the infected bone site 15 days post‐treatment. H) 3D micro‐CT images of the infected bone site 30 days post‐treatment. I) Quantitative analysis of BV/TV, Tb.Th, Tb.Sp, and Tb.N was performed based on micro‐CT data (N = 3). J) H&E staining of the infected defect area at day 15. K) H&E staining of the infected defect area at day 30. L) Masson's trichrome staining of the infected defect area at day 15. (M) Masson's trichrome staining of the infected defect area at day 30.

The mechanistic diagram of capsules to treat osteomyelitis was demonstrated in **Figure** **7**A. Osteomyelitis model was constructed by infecting with *S. aureus* and treated with capsules (Figure 7B). After 15 days of treatment with capsules, the number of colonies in the control group, the PC and the PC‐O group was significantly higher than that in the PC@TOB and PC‐O@TOB groups, indicating that the capsules in these two groups had significant antibacterial effects at the infection site (Figure 7C; Figure S12, Supporting Information). Further CFU results after 30 days showed almost no colony growth in the PC@TOB and PC‐O@TOB groups (Figure 7D). It was due to the ability of the capsules to capture free *S. aureus* in the tibia and the presence of TOB in the capsules. After 15 days, compared with the control group, the PC‐O@TOB group shows significant bone healing effects, with the reduction of bone holes and inflammatory response in the bone at the infection site (Figure 7E,F). After 30 days, the bone surface tended to be smooth without obvious deformity. These results indicated that the PC‐O@TOB capsules could effectively control infection and significantly promoted the repair and regeneration of bone tissue. Micro‐computed tomography (Micro‐CT) analysis further revealed the impact of different capsules on bone healing. The PC‐O@TOB group exhibited better bone healing effect than that of other groups, showing higher bone structural integrity and a relatively smooth surface after 15 days of treatment (Figure 7G), whereas the PC‐O@TOB group showed good bone regeneration after 30 days of treatment with virtually no holes in the bone surfaces (Figure 7H). Based on the quantitative analysis results of micro‐CT (Figure 7I), the PC‐O@TOB group showed significant advantages in trabecular bone repair, especially in key parameters of bone volume fraction (BV/TV), trabecular thickness (Tb.Th), trabecular spacing (Tb.Sp) and trabecular number (Tb.N). It indicated that the PC‐O@TOB group had a higher proportion of bone tissue volume generated with thicker and more tightly distributed trabeculae, further confirming its potential in promoting bone structure reconstruction. Overall, the PC‐O@TOB capsule provided a comprehensive and effective treatment strategy for bone repair after osteomyelitis through synergistic antibacterial, anti‐inflammatory, antioxidant and osteogenesis‐promoting effects.

Hematoxylin and eosin (H&E) staining results provided more visual evidence for the superiority of PC‐O@TOB capsules in the treatment of osteomyelitis. The H&E staining on day 15 showed that bone tissue regeneration was significantly enhanced in the PC‐O@TOB group, with a larger area of newly formed bone (Figure 7J,K). The infiltration of inflammatory cells in this group was also reduced, indicating that the capsules not only effectively inhibited infection but also promoted early tissue repair (Figure S13, Supporting Information). In contrast, the control group and other capsule treatment groups showed less obvious bone regeneration and persistent inflammation. Masson's trichrome staining (Figure 7L,M) further confirmed these findings. The blue‐stained areas in the images reflect the collagen deposition, indicating early bone formation and mineralization. The healing effect was more pronounced in the PC‐O@TOB group after 30 days of treatment. Compared with healthy rat tibias, the overall integrity and structure of the bone were basically the same as those of healthy rat tibias after treatment with PC‐O@TOB (Figure S14, Supporting Information). The improved bone density and structure in the PC‐O@TOB group highlighted the capsule's ability to promote the regeneration of a strong and healthy bone matrix, which was a key result for the successful treatment of osteomyelitis.

### 2.8 Evaluation of Osteogenesis, Immune Responses, Angiogenesis, and Transcriptome Analysis During the Osteogenic Process

**Figure 8:**
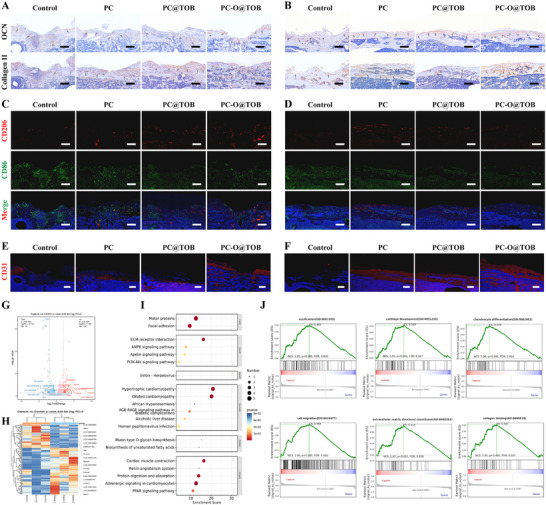
Comprehensive evaluation of osteogenesis, immune modulation, angiogenesis, and transcriptome dynamics in osteomyelitis. IHC of OCN and COL2 in the infected defect area A) at 15 days and B) at 30 days. Scale bars are 500 µm. Immunofluorescent staining of CD206 (red) and CD86 (green) in the infected defect area with nuclei counterstained in blue C) at 15 days and D) at 30 days. Scale bars are 500 µm. IF of CD31 (red) in the infected defect area E) at 15 days and F) at 30 days. Scale bars remain 500 µm. G, H) Transcriptome heatmaps that display the (DEGs of the PC‐O@TOB group compared with the control group, with both upregulated and downregulated genes shown. I) KEGG pathway enrichment analysis that compares the top 20 enriched pathways (categories: Environmental Information Processing and Cellular Processes) between the PC‐O@TOB group and the control group. J) GSEA of gene sets related to ossification, cartilage development, chondrocyte differentiation, cell migration, extracellular matrix structural constituents, and collagen binding.

In the osteomyelitis‐infected defect area, OCN immunohistochemistry (IHC) revealed positive expression regions (as indicated by arrows). In infected control groups, local inflammation and pathological factors disrupted normal protein expression patterns, leading to aberrant bone structure (**Figure** **8**A). Notably, OCN and type II collagen (COL2) expression levels were markedly reduced in controls. However, the PC‐O@TOB capsule group exhibited substantially elevated OCN expression with basal‐to‐apical distribution, suggesting directional bone growth from the defect base. In particular, the expression of OCN in the PC‐O@TOB capsule group was significantly enhanced and approached the bone surface compared with the control group at day 30, suggesting that bone healing had entered the later stage. The expression level of the PC‐O@TOB capsule group was higher than that of other groups at day 15 from the immunohistochemical staining results of COL2. As the main component of cartilage matrix, COL2 plays an important role in the formation and maintenance of cartilage. The PC‐O@TOB capsule group showed a higher degree of osseointegration and the expression of COL2 was mainly concentrated in the incompletely repaired areas, which further demonstrated its effectiveness in the bone healing process at day 30 (Figure 8B). H&E staining revealed comparable organ morphology (heart, liver, spleen, lungs, and kidneys) between experimental and control groups, with no apparent treatment‐related lesions (Figure S15, Supporting Information).

Immunofluorescence staining (IF) analysis of CD206⁺ (M2 macrophages, red) and CD86⁺ (macrophages, green) demonstrated attenuated green fluorescence in PC, PC@TOB, and particularly PC‐O@TOB groups versus controls, indicating reduced macrophage infiltration and inflammation (Figure 8C; Figure S16A, Supporting Information). Concomitantly, upregulated CD206 expression suggested effective M2 polarization induction by capsule formulations. In particular, the PC‐O@TOB capsule group had constructed an immune microenvironment conducive to tissue repair in the early stage through the antibacterial effect of TOB and OGP promoting bone repair by regulating bone immune responses and inhibiting the inflammatory response of M1 macrophages (Figure 8D; Figure S16B, Supporting Information). Angiogenesis analysis via CD31⁺ staining (red) revealed significantly enhanced vascularization in PC‐O@TOB groups at both 15 and 30 days, confirming its therapeutic advantage in late‐stage bone repair (Figure 8E,F; Figure S16C, D, Supporting Information). These findings collectively demonstrate the capacity of PC‐O@TOB capsules to orchestrate bone repair through coupled immunomodulation and angiogenic promotion.

Differentially expressed genes (DEGs) analysis revealed that the PC‐O@TOB capsule group exhibited 104 upregulated and 52 downregulated genes compared to the control group (FDR‐adjusted *p*‐value < 0.05) (Figure 8G–H). Gene Ontology (GO) enrichment analysis of upregulated DEGs (Figure S17, Supporting Information), suggests that PC‐O@TOB modulates critical biological processes to promote severe tibia infection repair in rats. These biological processes include “Negative regulation of osteoblast differentiation”, which maintains the balance of bone remodeling by inhibiting excessive differentiation of osteoblasts; “Cartilage development” – driving cartilage tissue reconstruction, a pivotal process for healing infection‐induced skeletal damage; and “Extracellular matrix (ECM) organization” stabilizing tissue architecture via ECM remodeling to enhance cellular adhesion and structural support for bone regeneration. Additionally, PC‐O@TOB potentiated immune responses via “Myeloid dendritic cell activation”, bridging innate and adaptive immunity to accelerate infection containment. This immunomodulatory effect likely establishes a pro‐regenerative microenvironment for bone healing. Collectively, PC‐O@TOB facilitates multimodal therapeutic outcomes: simultaneously promoting bone repair/regeneration and orchestrating immune‐cell signaling crosstalk through osteoblast differentiation control, cartilage remodeling, ECM reorganization, and dendritic cell activation, thereby comprehensively enhancing recovery from osteomyelitis.

KEGG pathway enrichment analysis identified significant modulation of the following key pathways by PC‐O@TOB (Figure 8I; Figure S18, Supporting Information). First, in the extracellular matrix (ECM)‐receptor interaction pathway, the PC‐O@TOB capsule may promote tissue repair after osteomyelitis by regulating the structure and function of the ECM. Second, the AMPK signaling pathway plays an important role in cellular energy metabolism, reducing inflammation, and promoting cell survival. In addition, the PI3K‐Akt signaling pathway is closely related to cell survival and growth. In gene set enrichment analysis (GSEA) analysis (Figure 8J), gene sets related to bone morphogenesis (ossification, cartilage development, chondrocyte differentiation), cell migration, ECM structural integrity, and collagen binding in the PC‐O@TOB group. Pathway‐level insights suggest that PC‐O@TOB synergistically enhances bone repair via coordinated cell migration and ECM stabilization, while concurrently addressing metabolic dysregulation, vascularization, and tissue recovery in osteomyelitis.

## 3 Conclusion

In conclusion, we have engineered a multifunctional PC‐O@TOB capsule that effectively captures and eliminates bacteria while combining anti‐inflammatory, immunomodulatory, and oxidative stress alleviation properties. In a rat osteomyelitis model, PC‐O@TOB capsules exhibited pronounced antibacterial efficacy, stimulated bone tissue regeneration, attenuated inflammatory responses, modulated immune activity, and promoted angiogenesis. Transcriptomic analysis demonstrated that PC‐O@TOB capsules optimize extracellular matrix (ECM) structure, activate the AMPK signaling pathway to enhance mitochondrial metabolism in osteoblasts, and boost PI3K‐Akt pathway activity (RUNX2/BMP2‐mediated) to drive bone cell proliferation and differentiation. Importantly, this study promotes bone regeneration by modulating the osteoimmune microenvironment, thereby providing a new perspective for therapeutic strategies targeting osteomyelitis.

## 4 Experimental Section

### Reagents

Calcium chloride anhydrous (CaCl_2_, 96.0%), sodium carbonate anhydrous (Na_2_CO_3_, ≥ 99.8%), procyanidins (PC), tobramycin (TOB), hydrochloric acid (HCl), hydrogen peroxide solution (H_2_O_2_), and xylenol orange indicator were purchased from Aladdin Industrial Corporation (Shanghai, China). K6‐OGP (KKKKKK‐GGYGFGGYGFGG, MW = 11 864.18) polypeptides were synthesized by Nanjing Peptide Valley Biotechnology Co., Ltd (Nanjing, China). Cell culture reagents including fetal bovine serum (FBS), Dulbecco's modified Eagle's medium (DMEM), and Minimum Essential Medium Eagle (MEM) were from Gibco. Cell counting kit‐8 (CCK‐8) reagents were purchased from Dojindo Molecular Technologies (Shanghai, China). DPPH radical scavenging capacity assay kit, ABTS radical scavenging capacity assay kit, 2,7‐dichlorodihydrofluorescein diacetate (DCFH‐DA), 4′,6‐diamidino‐2‐phenylindole dihydrochloride (DAPI, ≥ 90%) were obtained from Solarbio Science Life Science (Beijing, China). Lipopolysaccharide (LPS), and Streptozotocin were purchased from Sigma‐Aldrich (Shanghai, China). Anti‐mouse ELISA kits for IL‐6, IL‐10, VEGF, IL‐β, TGF‐β and TNF‐α were obtained from Multisciences, and BMP‐2 was purchased from Cloud Clone Corp. Cell culture flask (TCF011025), cell culture dish (TCP010006, TCP010024, and TCP010096) and PCR tubes (PCR520200) were purchased from Guangzhou Jet Bio‐Filtration Co., Ltd. GelNest Matrix Gel (211252), Glass‐bottom cell culture plates (801002 and 801004) and frozen storage tube (612521) were purchased from NEST Biotechnology Co. Ltd. (Wuxi, China). All reagents and materials were used without further purification.

### Synthesis of PC Capsules, PC‐O Capsules, PC@TOB Capsules, and PC‐O@TOB Capsules

600 mg of PC was dissolved in deionised water (10 mL), then the pH was adjusted to 9 by adding 2 M NaOH. Next, 3 mL of calcium chloride solution at a concentration of 0.33 M was injected into a conical flask, 2 mL of sodium carbonate solution at a concentration of 0.5 M and 1 mL of PC solution at a concentration of 60 mg/mL were added sequentially to this solution. The mixture was stirred continuously at 1200 rpm for 40 s and then allowed to stand for 15 min, followed by centrifugation at 1000 rpm for 3 min and washing with deionized water to remove impurities. After washing, PC capsules were prepared by adding 1 M HCl.

In order to determine the optimal pH condition, we conducted a verification experiment using TOB as a representative. Specifically, 1 mL of PC capsules (1 mg mL^−1^) were reacted with a slight excess of TOB (1.5 mg) under three pH conditions (6.0, 8.0, and 10.0). It was determined that the maximum loading was achieved under the condition of pH 8.0.

The method for preparing PC‐O and PC@TOB capsules: Take 1 mL of PC capsule solution (2 mg mL^−1^) and mix it with K6‐OGP or TOB to a final concentration of 1 mg mL^−1^, respectively. Adjust the system pH to 8.0 and stir at room temperature for 1 h. Unbound components were removed by five cycles of centrifugal washing (3000 rpm, 5 min each), yielding K6‐OGP‐loaded PC‐O capsules and TOB‐loaded PC@TOB capsules. For the second step, take 1 mL of PC‐O capsule solution (2 mg mL^−1^), mix it with both K6‐OGP and TOB to a final concentration of 1 mg mL^−1^, adjust the pH to 8.0, and stir at room temperature for 1 h. Finally, wash with deionized water through five centrifugal cycles (3000 rpm, 5 min each) to obtain dual‐loaded PC‐O@TOB capsules.

### Characterization

The morphological features of the microcapsules were characterized using a scanning electron microscope (SEM, Hitachi SU8010) and a transmission electron microscope (TEM, FEI Talos F200S). Confocal laser scanning microscope (CLSM) mounted on a Nikon A1 instrument was used to study micrographs of capsules and cells. Optical microscopy images were taken using a NIKON Ni‐U microscope and a 10 × lens bright field. A Fourier transform infrared spectrometer (Bruker TENSOR II) was used to measure the infrared spectra of the capsules and the materials used to prepare the capsules. X‐ray photon spectra (XPS) were obtained on a Thermo‐Electron ESCALAB 250 spectrometer. Sample absorbance was measured by a Lambda 25 spectrophotometer from PerkinElmer, USA. Enzyme‐linked immunoassay analyser (Tecan), flow cytometer (Becton‐Dickinson), Vortex mixer Bioland (China), Roche light cycler 480 fluorescence quantitative PCR instrument (Roche). The antioxidant capacity of capsules was assessed by the assay kit.

### Cell Culture

BMSCs derived from mouse bone marrow were obtained from Procell Life Science & Technology Co., Ltd. They were cultured at 37 °C in a 5% carbon dioxide atmosphere in α‐MEM basal medium supplemented with 100 U mL^−1^ penicillin, 100 mg mL^−1^ streptomycin, and 10% FBS. HUVECs and RAW 264.7 and obtained from Procell Life Science & Technology Co., Ltd, which were cultured at 37 °C in a 5% carbon dioxide atmosphere in DMEM basal medium supplemented with 100 U mL^−1^ penicillin, 100 mg mL^−1^ streptomycin, and 10% FBS.

### ROS Scavenging Capacity of the Capsule In Vitro

The 5 × 10^4^ cells per well were inoculated into 24‐well plates with coated glass plates. At the end of co‐culture with different capsules, the cells were stained with Calcein‐AM/PI (CA1630, Solarbio) and cell viability was observed by laser confocal scanning microscopy. Viability was additionally quantified using a CCK‐8 assay. Then the internalization of capsules was observed using CLSM. The DCFH‐DA probe was used to detect intracellular ROS levels. Flow cytometry assay and CLSM images were used to observe the intracellular fluorescence signal.

### Osteogenic Differentiation Assay

ALP activity and ARS staining were used as early and late markers of osteogenic differentiation, respectively. After 14 days of osteogenic induction, ALP activity was assessed by staining using the BCIP/NBT Alkaline Phosphatase Colouring Kit (Biotronik) and ALP Detection Kit (Biotronik) according to the manufacturer's instructions. To assess late mineralization, cells were fixed with 4% paraformaldehyde after 21 days in culture and then incubated with 0.1% Alizarin Red S solution (pH 4.2) for 30 min. After 14 days of culture as above, RNA from BMSCs was extracted with TRIzol reagent and reverse transcribed into cDNA using the PrimeScript RT kit. Table S1 (Supporting Information) lists the ortho‐ and reverse‐primers for osteogenesis‐related genes. Data were analyzed using the 2^−ΔΔct^ method and normalized to the mean of the control group. After being observed and photographed with a microscope, calcium deposits were extracted using 10% cetylpyridinium chloride solution for 1 h and were quantified by measuring absorbance at 593 nm.

### Macrophage Phenotype Regulation In Vitro

RAW 264.7 cells (5 × 10^5^ cells per well) were seeded in 6‐well plates and polarized into pro‐inflammatory M1 macrophages using 100 ng mL^−1^ LPS and 20 ng mL^−1^ IFN‐γ. Surface markers were analyzed via fluorescence‐activated cell sorting (FACS): cells were incubated with FITC‐conjugated anti‐CD86 (M1) and APC‐conjugated anti‐CD206 (M2) antibodies (BioLegend, USA) for 30 min at 4 °C. Flow cytometry (CytoFLEX, Beckman Coulter, USA) and CytExpert software were used for data acquisition and analysis.

### ELISA

RAW 264.7 cells (5 × 10^4^ cells per well) were pretreated with capsules for 24 h, then stimulated with LPS (100 ng mL^−1^) and IFN‐γ (20 ng mL^−1^) for an additional 24 h. Subsequently, the cells were further cultured in an environment containing 100 ng mL^−1^ LPS and 20 ng mL^−1^ INF‐γ for another 24 h. To obtain the cell supernatants, centrifugation was performed at 3000 revolutions per minute for 15 min at 2 – 8 °C. The obtained supernatants were immediately used for subsequent experiments. Finally, the contents of various growth factors in the supernatant, including TNF‐α, IL‐6, IL‐1β, IFN‐γ, IL‐10, TGF‐β, BMP‐2, and VEGF, were determined by ELISA, and the cells treated with LPS and INF‐γ were used as positive control.

### Bacterial Culture

Bacterial culture experiments were as follows: *S. aureus*, *P. aeruginosa* and *E. coli* strains were purchased from American Standard Culture Collection (ATCC). Both strains were inoculated on Luria‐Bertani (LB) agar plates manufactured by Hangzhou Microbiology Reagent Co. First, one colony was picked from the plate and inoculated into a test tube containing 10 mL of appropriate liquid growth medium LB, and then incubated at 37° C for 24 h. Then, the bacterial cells were collected by centrifugation (5000 × g, 5 min) and the bacterial suspension was diluted with phosphate buffer solution (PBS) (PBS or growth medium was used as a diluent) to the specific concentration required for the experiment. Finally, the final concentration of the bacterial suspension was accurately determined by plate counting.

### Anti‐Bacterial Assays

The different types of capsules were immersed in 10 mL of LB medium containing 10^6^ CFU mL‐1 of bacteria for 24 h. Bacterial survival was calculated:

(1) $$Bacterial\;survival\;\left(\%\right)=\left\{\frac{\left(ODsample-\;ODblank\right)}{\left(ODcontrol-\;ODblank\right)}\right\}\times 100\%$$

OD_sample_ refers to media containing capsules co‐cultured with bacteria, OD_control_ refers to media containing only bacteria, and OD_blank_ refers to media without bacteria. Similarly, the co‐culture solution (50 µL) was incubated in sterile agar plates for 24 h to check for colony re‐formation.

Regarding bacterial morphology under SEM, first 2.5% pentylene glycol was incubated overnight at 4 °C; pbs washed three times for 10 min each; and dehydrated in 30%, 50%, 70%, 80%, 90%, 95%, and 100% ethanol solutions at 4 °C for 20 min, respectively. (3) Then the samples were washed three times with PBS. After the samples and controls were dried under vacuum (oven drying was actually used), they were sprayed with gold and subjected to SEM measurements.

Mature *S. aureus* was incubated with different types of capsules or PBS at 37 °C for 3, 6, and 12 h. The medium was then removed, stained with SYTO 9 and PI for 20 min in the dark, and then visualized with a laser scanning confocal microscope.

For the circle of inhibition experiment, first, *S. aureus* was evenly coated onto a solid plate, and then in fact, the same size of filter paper sheet was put on it, and then 5 µL of 1mg mL^−1^ of different types of capsules were added, and the plate was placed in the incubator at 37 °C for 18 h and then photographed for observation.

### In Vivo Antibacterial and Osteomyelitis Therapeutic Effect Evaluation

Twenty‐four Sprague‐Dawley (SD) rats weighing about 300 g and aged about 6 weeks were purchased from the Experimental Animal Centre of Hangzhou Medical College. They were housed in an SPF environment at the Experimental Animal Centre of Wenzhou Research Institute, University of Chinese Academy of Sciences, with sufficient water and food, 12 h of light and 12 h of darkness in a circadian rhythm. All animal experimental procedures were conducted in strict accordance with the Guidelines for the Breeding and Use of Laboratory Animals of Wenzhou Research Institute, University of Chinese Academy of Sciences, and approved by the Animal Ethics Committee of Wenzhou Research Institute, University of Chinese Academy of Sciences, with permission number WIUCAS24122301.

For the establishment of the infectious bone defect model, rats were anaesthetized effectively with sodium pentobarbital 1 mg kg^−1^, the lower limbs of the rats were shaved, and the skin of the proximal part of the lower leg below the knee joint was incised. The subcutaneous and muscle tissues were further separated to fully expose the tibial plateau and the lower part of the plateau. After drilling holes in the upper end of the tibial plateau with an orthopaedic microdrill with a diameter of 1 mm, the bone marrow cavity was injected with 50 µL of *S. aureus* (concentration of 10^7^ CFU mL^−1^) for modelling. After the bone wax was allowed to fill the bone defect, the surgical wound was sutured and the osteomyelitis model was successfully constructed 7 days later. All surgical sites were re‐incised, debridement procedures were performed, and the trauma was rinsed with a large amount of PBS, and then 50 µL of PC group, PC@TOB group, and PC‐O@TOB at a concentration of 1 mg mL^−1^ was injected into the bone marrow cavity of the model, and 50 µL of PBS was injected into the control group. In order to check for therapeutic efficacy, after lethal euthanasia by injection of sodium pentobarbital on 15 and 30 days, respectively, and in order to determine the antimicrobial activity, the bone infection site was rinsed and collected by injecting 100 µL PBS with a pipette gun, and the suspension was inoculated on LB) agar plates and incubated at 37 °C for 24 h. The suspension was then incubated with LB agar plates for 24 h at 37 °C. Meanwhile, the harvested rat tibia pins were fixed in 4% paraformaldehyde, decalcified in 10% ethylenediaminetetraacetic acid (EDTA) solution, dehydrated in increasing concentrations of ethanol, and finally embedded in paraffin.

### Microcomputed Tomography Analysis

The distal femur was photographed using micro‐CT with the following parameters: voltage 80 kV; current 300 mA; and rotation 360° with a rotation step of 0.5°. Regions of bone infection/defects were emphasized during 3D reconstruction using CTAn and CTVol software (Bruker Corporation). Bone morphometric parameters, including BV/TV, Tb.Th, Tb.N, and Tb.Sp, were calculated.

### In Vivo Biosafety Assessment

According to the protocol, 30 days after capsule treatment, the major internal organs of mice, including the heart, liver, spleen, lungs, and kidneys, were stained with eosin (H&E). The presence of bacterial residues in the organs and the safety of the bioactive material were determined.

### Histology and Histomorphometry

Decalcified, paraffin‐embedded sections were prepared for H&E and Masson's trichrome staining to assess bone regeneration. IHC was performed for OCN and COL2. Additional IF staining targeted CD31 (endothelial marker), CD206 (anti‐inflammatory macrophage marker), and CD86 (pan‐macrophage marker).

### Transcriptome Analysis

Total RNA was extracted from the bone infected area of the tibia in the osteomyelitis model using Trizol reagent according to the manufacturer`s protocol. RNA purity and quantification were evaluated using the NanoDrop 2000 spectrophotometer (Thermo Scientific, USA). RNA integrity was assessed by Agilent 2100 Bioanalyzer (Agilent Technologies, USA). The libraries were constructed using VAHTS Universal V6 RNA‐seq Library Prep Kit according to the manufacturer`s instructions. The transcriptome sequencing and analysis were conducted by OE Biotech Co., Ltd. (Shanghai, China).

### Statistical Analysis

All quantitative data are expressed as the mean ± standard error (SD). The statistical differences between groups were determined using one‐way ANOVA followed by Tukey's test analysis (GraphPad Prism version 5). A statistically significant difference was considered at a minimal level of significance of *p* < 0.05, and denoted as *p < 0.05, **p < 0.01, ***p < 0.001, ****p < 0.0001.

## Conflict of Interest

The authors declare no conflict of interest.

## Author Contributions

D.Y. and C.S. contributed equally to this work. D.Y., C.S., and S.W. investigated, designed the study, and wrote the original manuscript. D.Y. and C.X. performed most experiments. D.Y. analyzed data with intellectual contributions. X.Z. and L.L. revised the manuscript. X.Z. supervised this work. C.W. and L. Li acquired the funding. All authors have read and approved the final manuscript for submission.

## Supporting information



Supporting Information

## Data Availability

The data that support the findings of this study are available from the corresponding author upon reasonable request.
