# Supplementary material for: Bacterial Capture‐Killing Capsules with Remodeling Bone Immune Microenvironment for the Effective Treatment of Osteomyelitis
Source: Adv Sci (Weinh). 2025 Apr 7;12(25):2501505. doi: 10.1002/advs.202501505 (PMC12224966; doi:10.1002/advs.202501505)
Supplement: Supplementary file 1 — Supporting Information [file ADVS-12-2501505-s001.docx]

**Bacterial Capture-killing Capsules with Remodeling Bone Immune Microenvironment for the Effective Treatment of** **Osteomyelitis**

Dong Yang^¶^ ^#^, Chang Shu^ф #^, Chengwei Xu^§^, Xingjie Zan^¶, ‡^, Shaoyin Wei^¶, ‡^ *, Chenglong Wang^Ѱ^ *, and Lianxin Li^Ѱ^ *

^¶^ School of Ophthalmology and Optometry, Eye Hospital, School of Biomedical Engineering, Wenzhou Medical University, Wenzhou, Zhejiang, 325035, China

^ф^ Wenzhou Medical University Yongkang First People's Hospital, Jinhua, Zhejiang, 321300, China

^§^ Department of Orthopedics, The First Affiliated Hospital of Wenzhou Medical University, Wenzhou, Zhejiang, 325000, China

^‡^ Wenzhou Institute, University of Chinese Academy of Sciences, Wenzhou, Zhejiang, 325001, China

^Ѱ^ Department of Orthopaedics Surgery, Shandong Provincial Hospital Affiliated to Shandong First Medical University, Jinan, Shandong, 250021, China

^#^ Equal contribution authors.

^*^ Corresponding authors.

Emails: shaoyinwei@mail.sdu.edu.cn (S. Wei); [wangchenglong1214@gmail.com](mailto:wangchenglong1214@gmail.com) (C. Wang); [lxli@email.sdfmu.edu.cn](mailto:lxli@email.sdfmu.edu.cn) (L. Li)


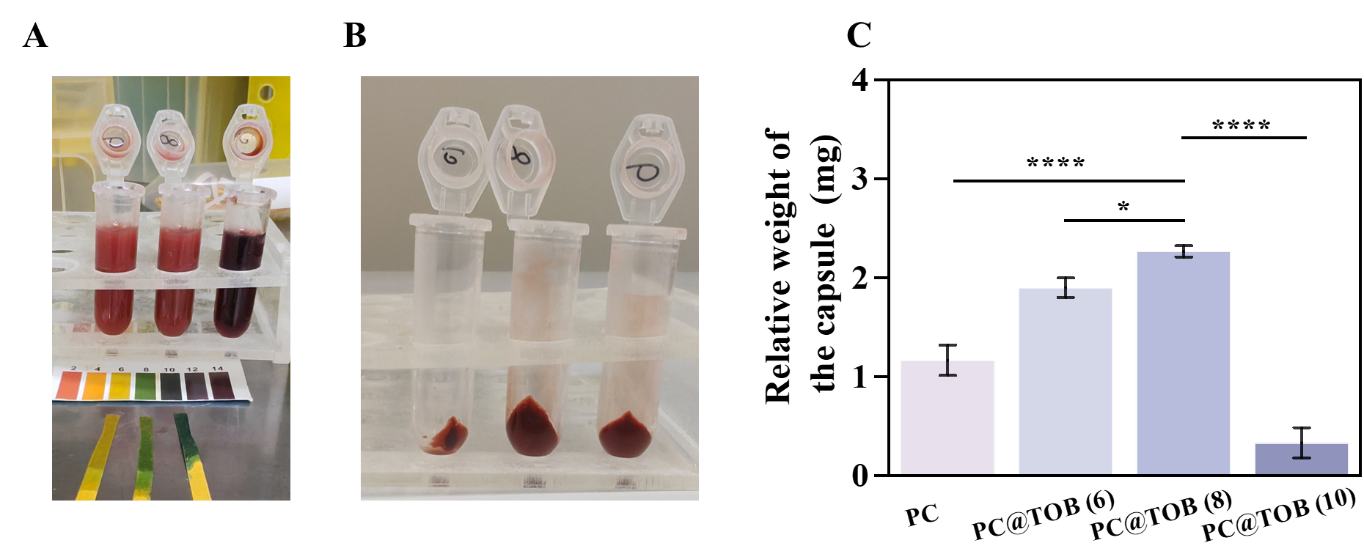


**Figure S1.** Interaction of PC capsules with tobramycin (TOB) under varying pH conditions. (A) Photographic documentation of capsules after pH adjustment and TOB addition. (B) Capsules post centrifugation and washing. (C) The results of quantitative analysis after freeze - drying the capsules.


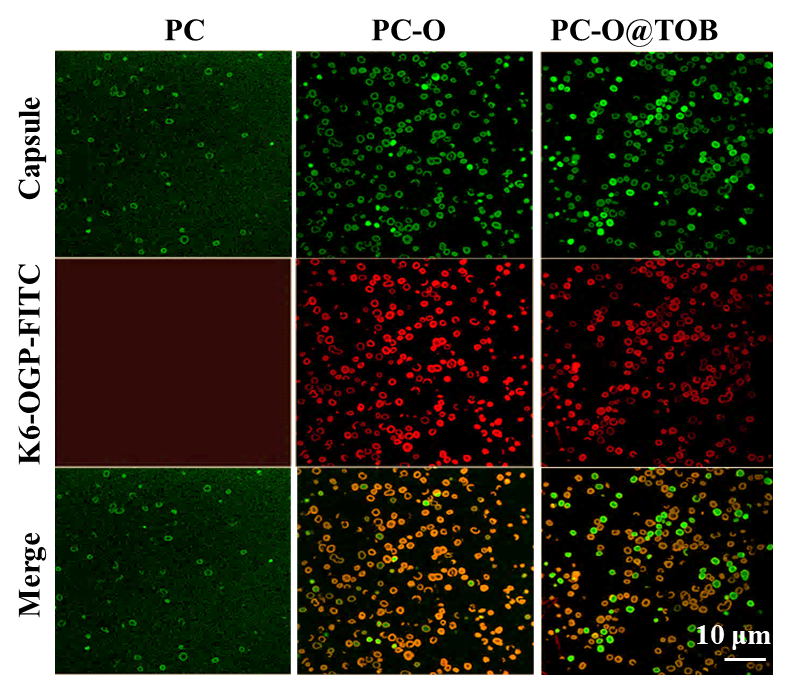


**Figure S2.** Confocal laser scanning microscope images of PC, PC-O, and PC-O@TOB capsules. The green color was the inherent fluorescence of PC capsules, and the red color was the fluorescence of TAMRA-K_6_-OGP.


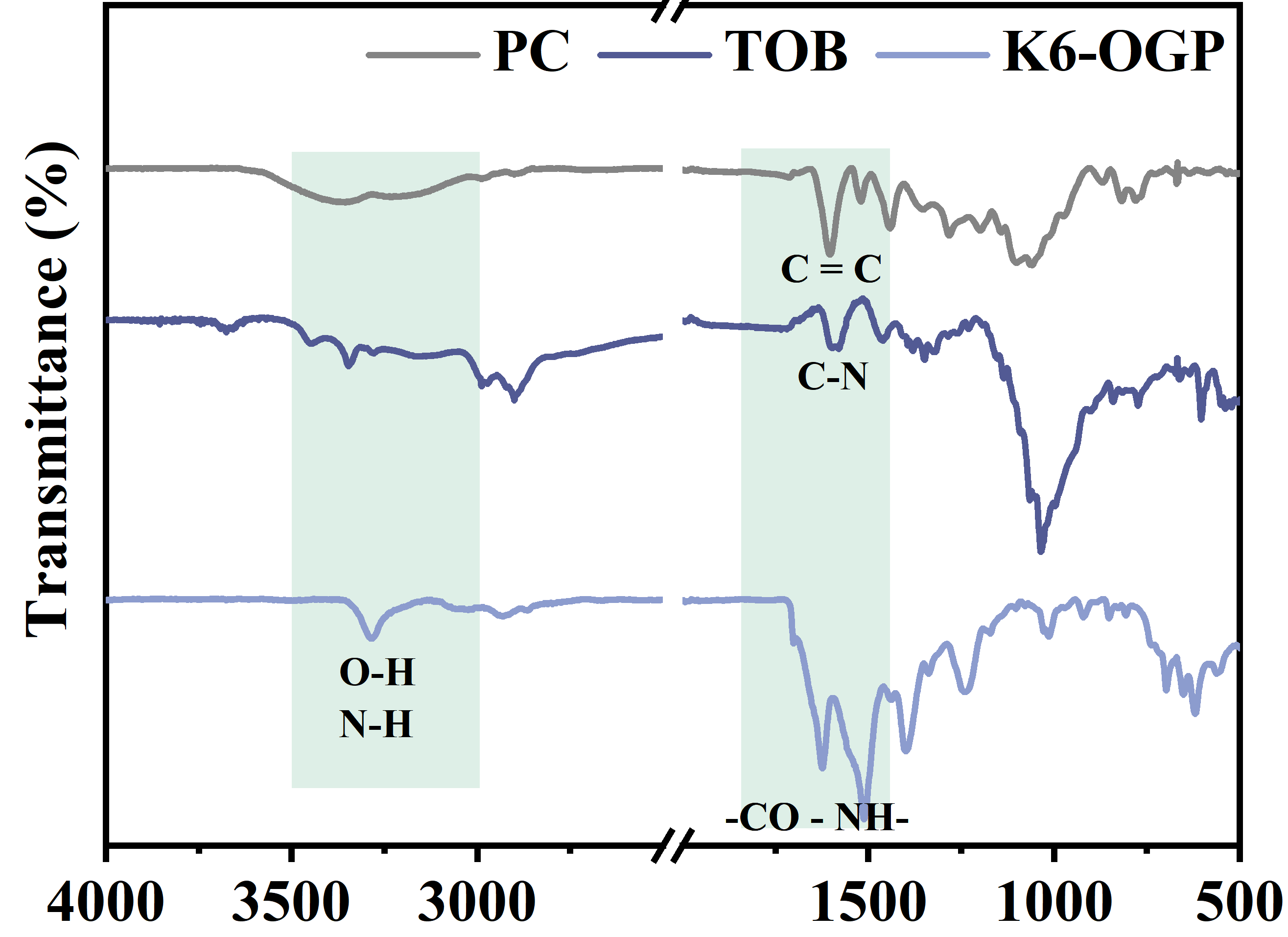
**Figure S3.** The FTIR analysis of PC, TOB, and K6-OGP fractions.


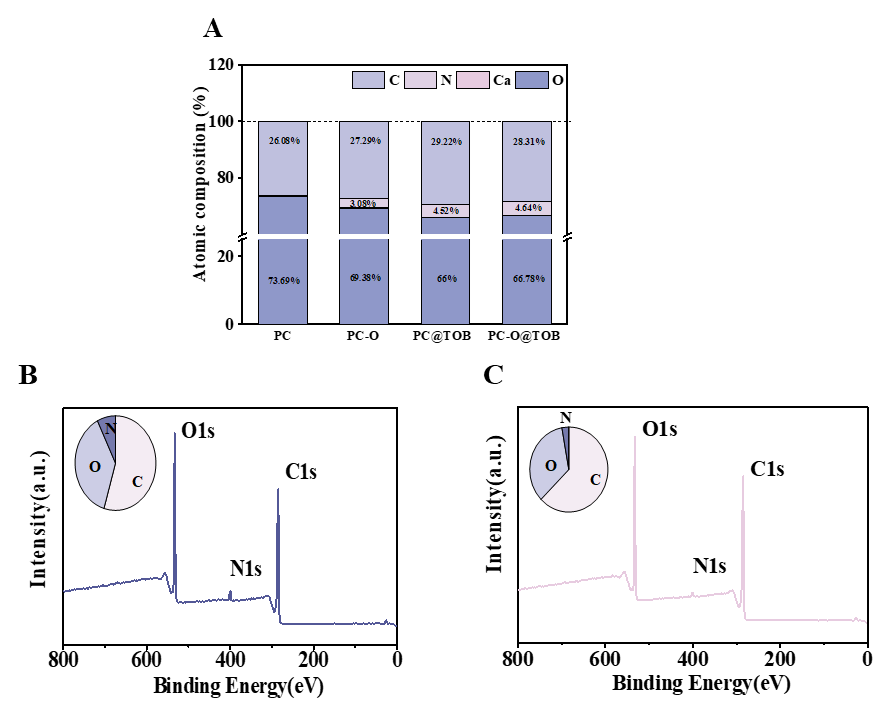


**Figure S4.** (A) Elemental content analysis of PC, PC-O and PC-O@TOB capsules by XPS. (B) XPS analysis of PC-O@TOB capsules after 3 days at pH 7.0 and (C) at pH 5.0.


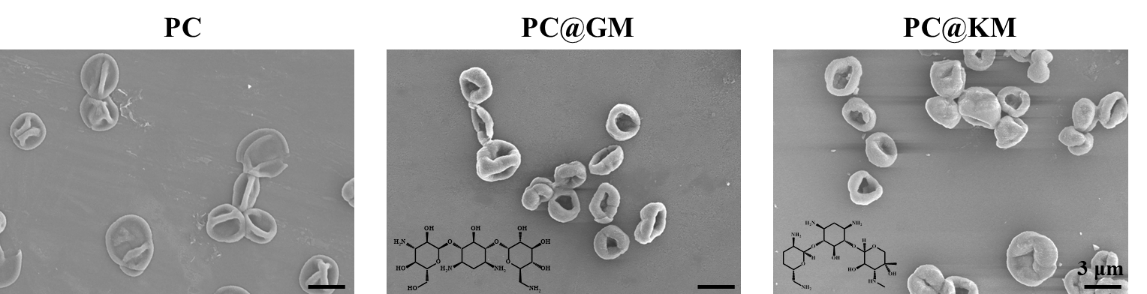


**Figure S5.** The images under SEM before and after the reaction of PC capsules with other aminoglycoside antibiotics.


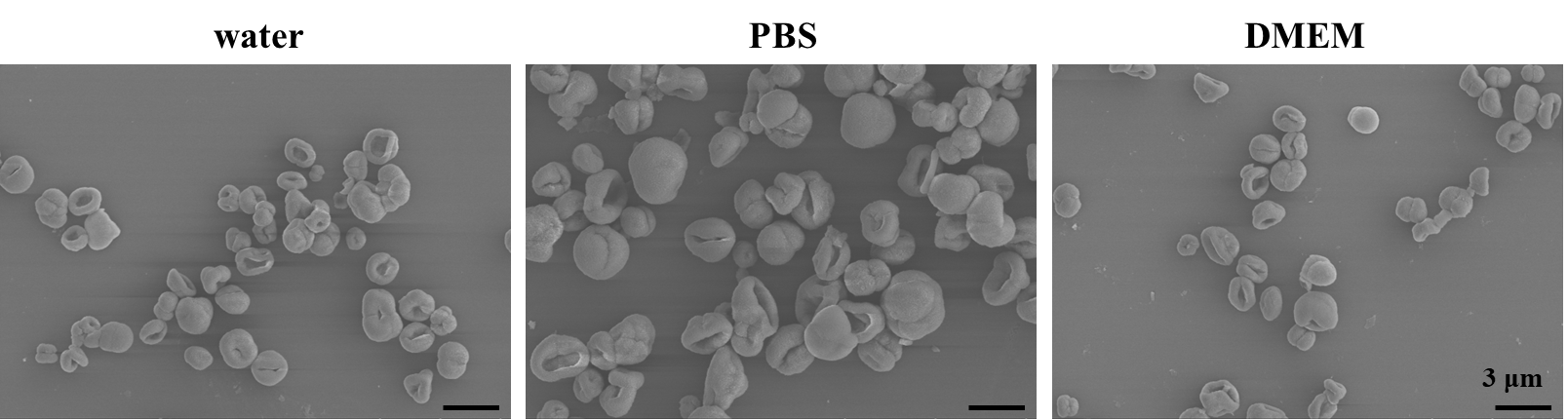


**Figure S6.** The images under SEM after treating PC-O@TOB capsules with water, PBS and DMEM for 3 days.

**Figure S7.** (A) Schematic of the proposed mechanism for polyphenol autoxidation within the capsule matrix. (B) DPPH radical scavenging activity of different capsule formulations over time. (C) Comparative efficiency of DPPH radical scavenging among the capsule formulations (N=4). (D) ABTS radical scavenging capacity of various capsule formulations (N=4).
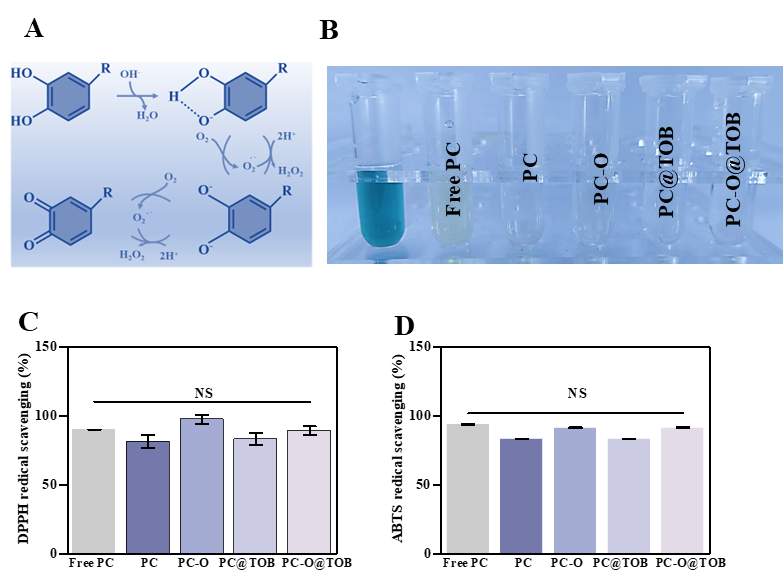


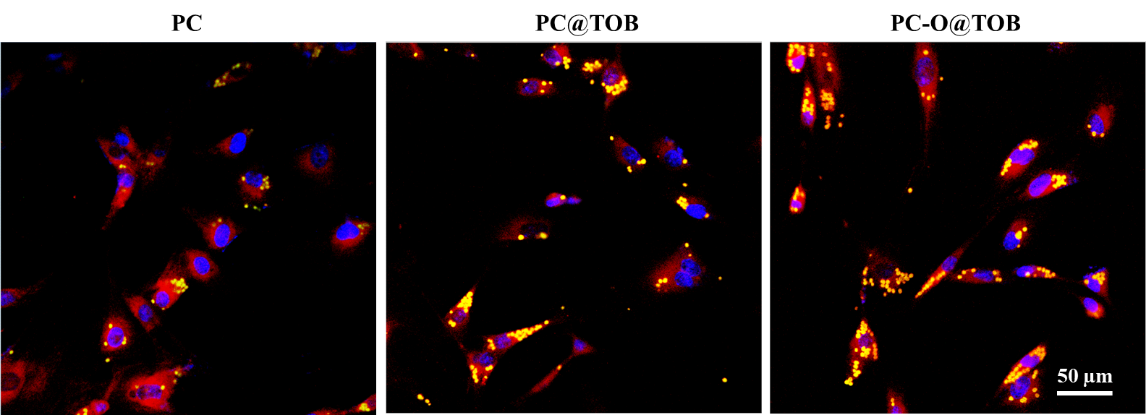


**Figure S8.** In the lysosome co-localization experiment, the nucleus is shown in blue, the lysosome is in red, and the yellow color represents the co-localization of the capsule within the lysosome.


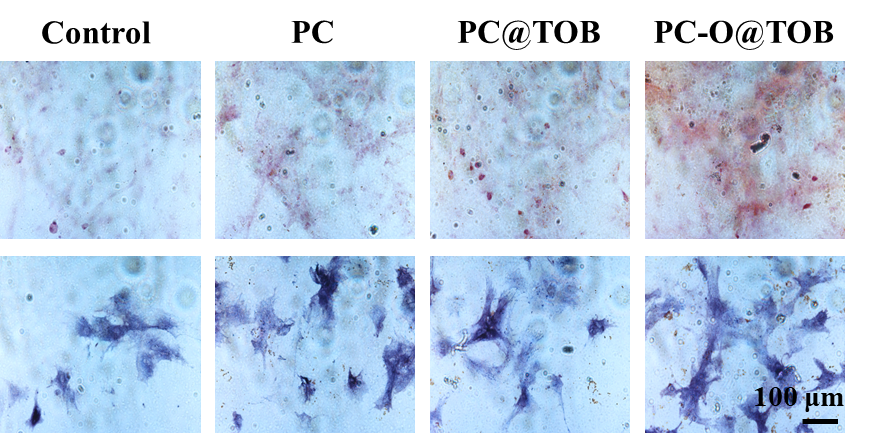


**Figure S9.** ALP staining and Alizarin Red S of BMMSCs cultured in osteogenic medium supplemented with different samples on day 7.


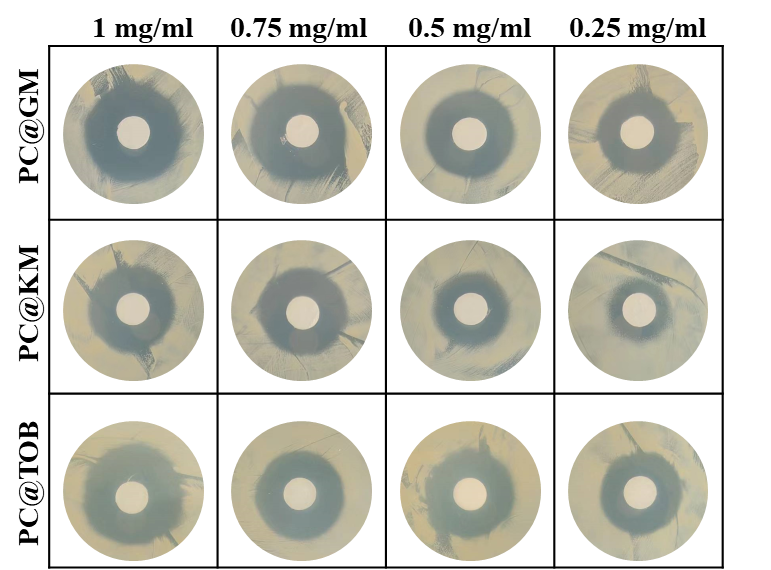


**Figure S10.** Bacteriostatic zones of PC@GM, PC@KM, and PC@TOB at different concentrations.


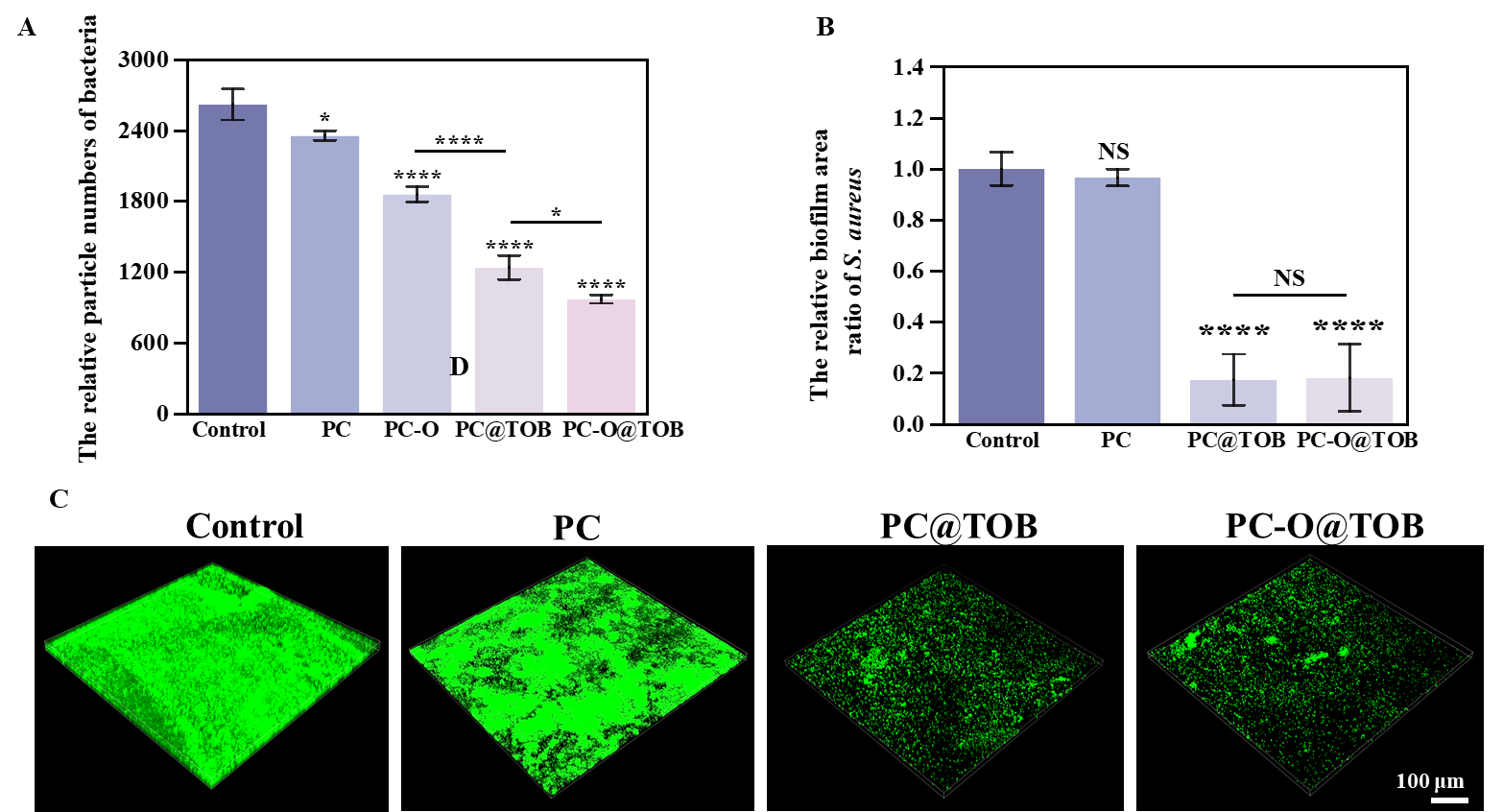


**Figure S11.** (A)The relative particle numbers of bacteria in different groups, including Control, PC, PC - O, PC@TOB, and PC - O@TOB. (B)Displays the statistical results of the relative biofilm area of S. aureus. (C) Shows fluorescence images of biofilms captured under a confocal microscope for the Control, PC, PC@T, and PC - groups.


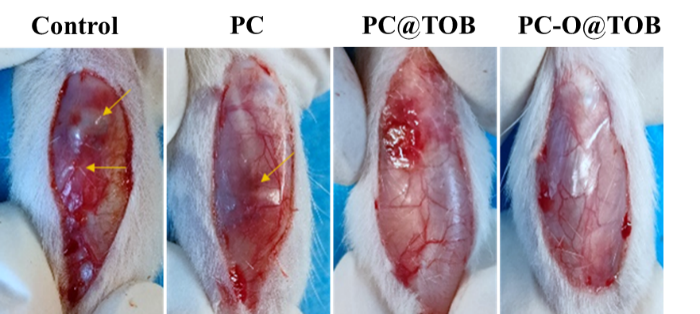
**Figure S12.** Photographs of incised rat calf epidermis at 15 days. Groups shown include Control, PC, PC@TOB, and PC-O@TOB


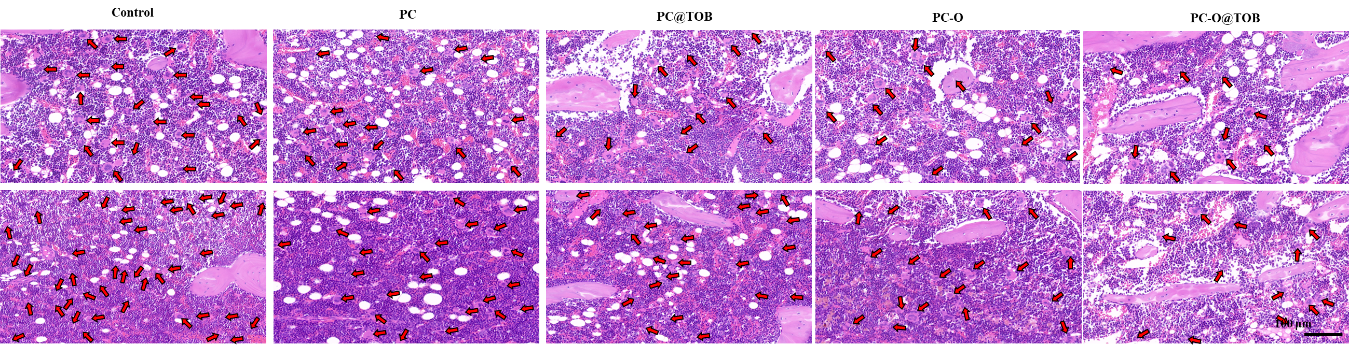


**Day 15**

**Day 30**

**Figure S13.** The local areas of the H&E staining in Figure 7 have been magnified, and the inflammatory cells are marked with red arrows.


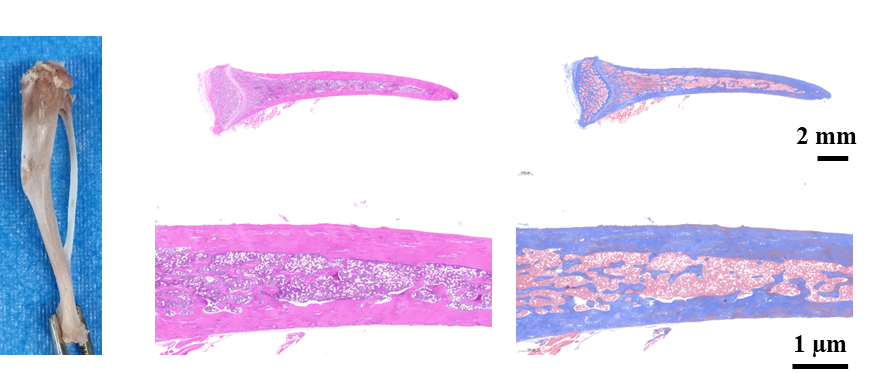
**Figure S14.** Pictures of tibiae of healthy rats, H&E and Masson stained.


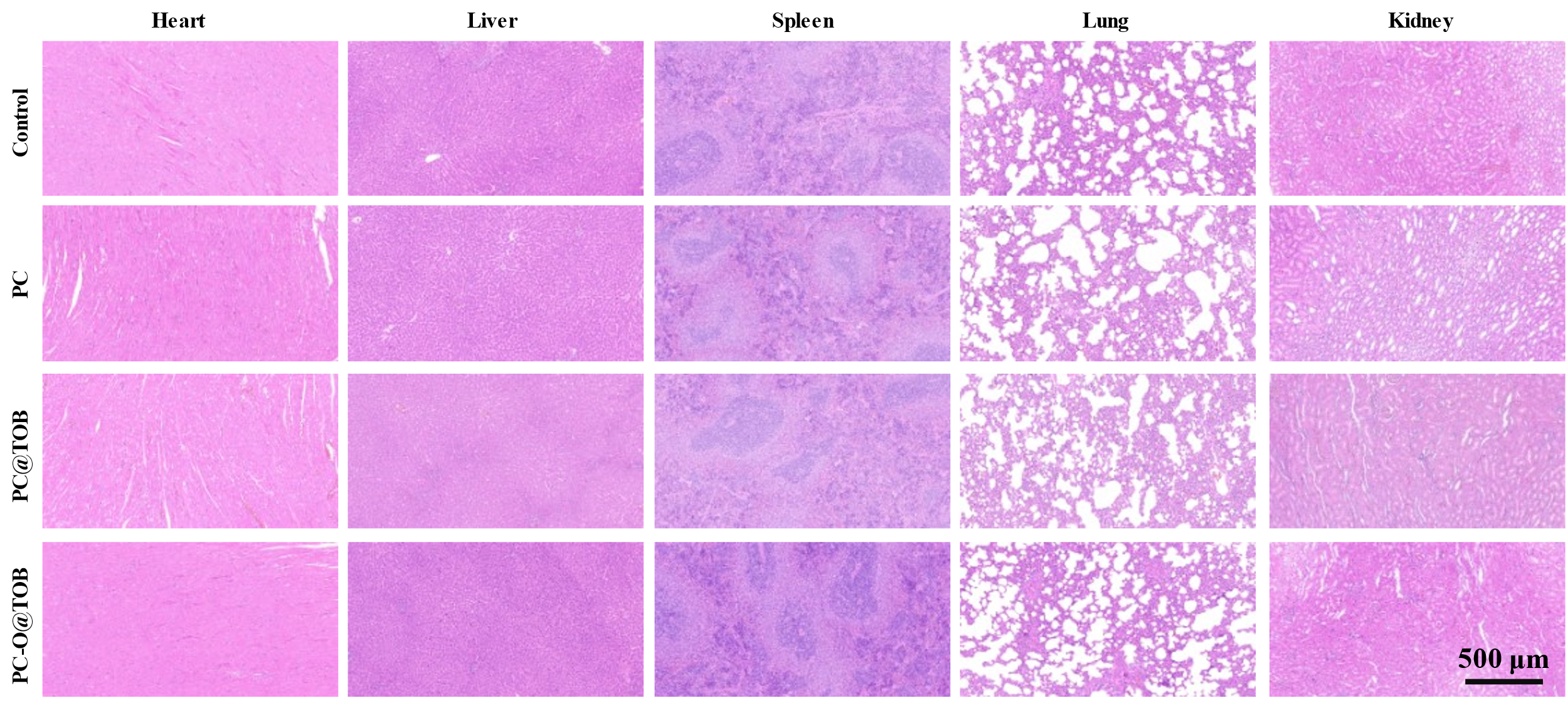


**Figure S15.** H&E staining of major rats organs.


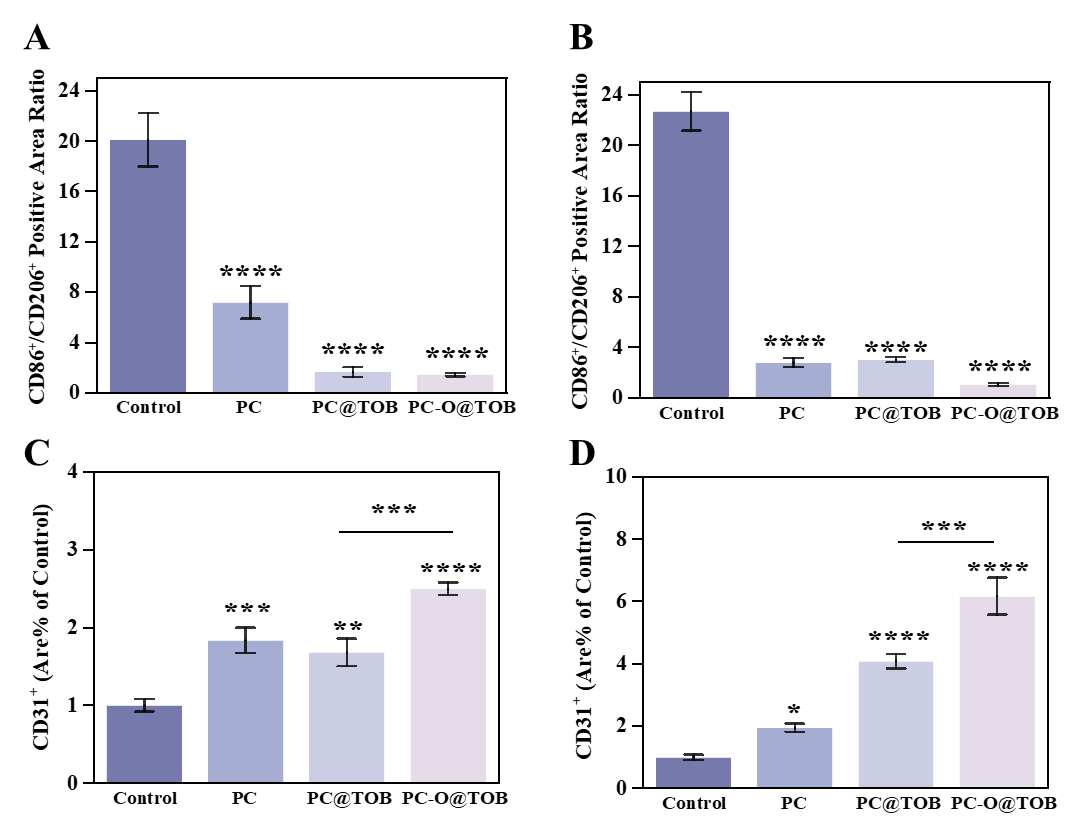


**Figure S16.** (A) and (C) respectively display the statistical of the fluorescence intensity of CD86⁺/CD206⁺ and CD31⁺ at 15 days. (B) and (D) respectively present the statistical results of the fluorescence intensity of CD86⁺/CD206⁺ and CD31⁺ at 30 days.


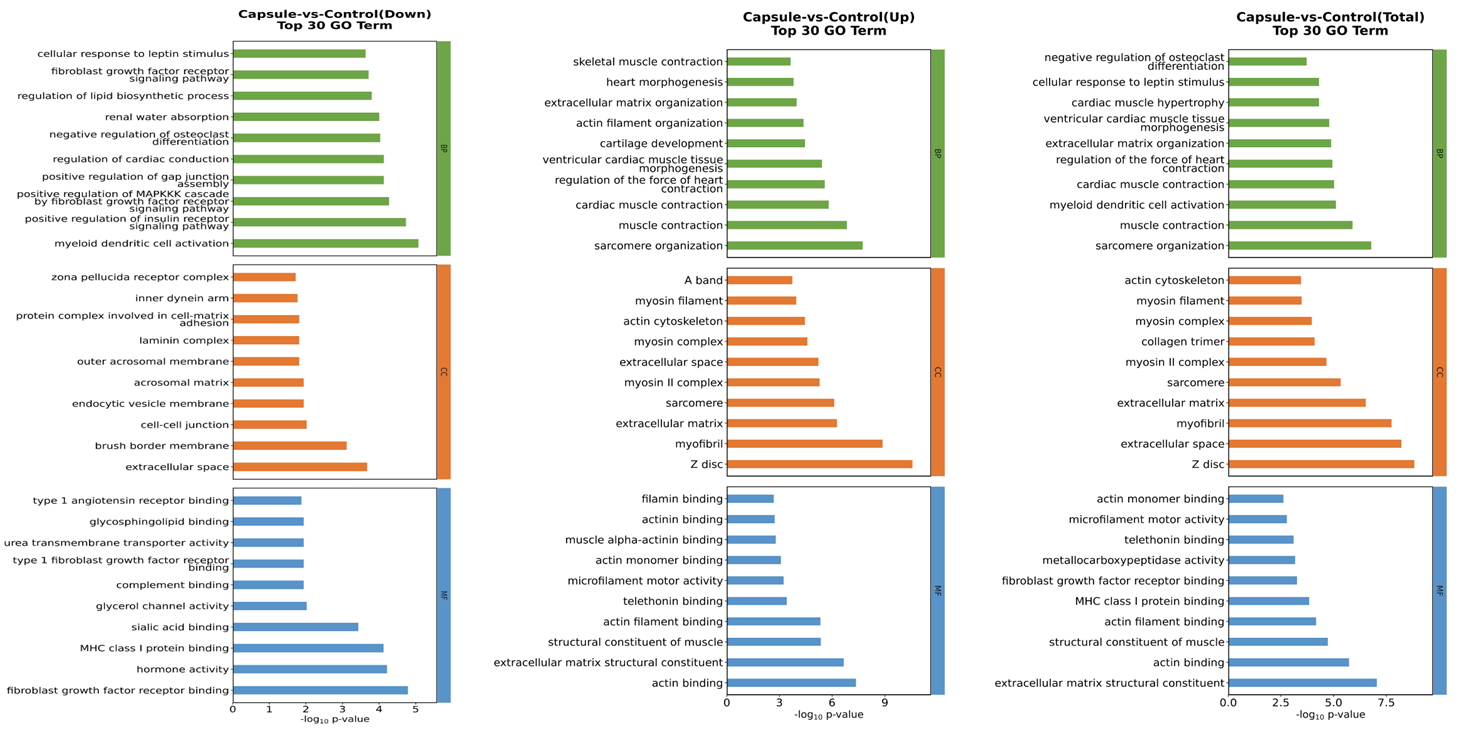
**Figure S17.** GO pathway enrichment analysis of DEGs with down-regulated、up-regulated and total-regulated expression.

**
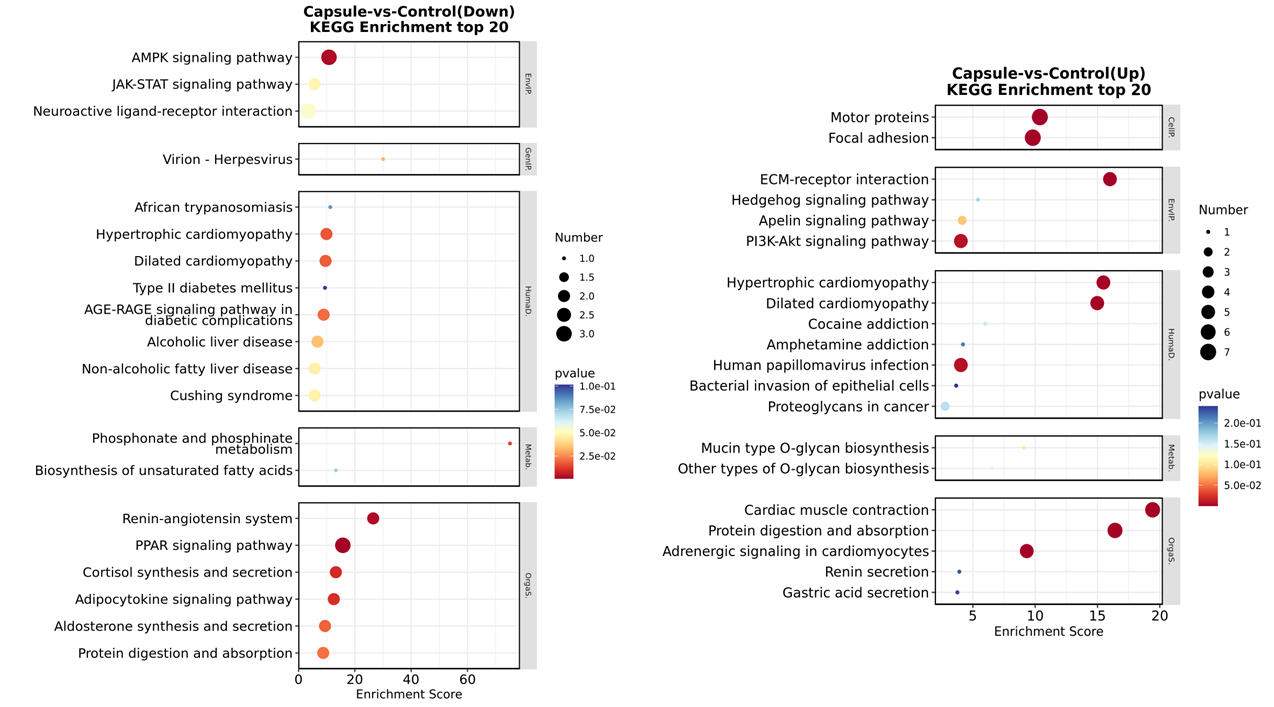
 Figure S18.** KEGG enrichment analysis of up-regulated and down-regulated genes.

**Table S1.** Primer sets (Rattus) used for quantitative real-time PCR.

| **RNA template** | **Forward primer (5′-3′)** | **Reverse primer (5′-3′)** |
| --- | --- | --- |
| β-Actin | CACCCGCGAGTACAACCTTC | CCCATACCCACCATCACACC |
| collagen I | CTGCCCAGAAGAATATGTATCACC | GAAGCAAAGTTTCCTCCAAGACC |
| OCN | GCCCTGACTGCATTCTGCCTCT | GGCTTGTCACTCGAATTTTGAGA |
| Runx2 | ATCCAGCCACCTTCACTTACACC | GGGACCATTGGGAACTGATAGG |
| ALP | TATGTCTGGAACCGCACTGAAC | CACTAGCAAGAAGAAGCCTTTGG |
